# Effect of vasopressin and its analogues on microcirculatory perfusion: a systematic review

**DOI:** 10.1186/s40635-026-00983-5

**Published:** 2026-10-10

**Authors:** Judith van Zwol, J. G. Kruijsse, A. A. Vidal Itriago, S. N. T. Hemmes, C. E. van den Brom

**Affiliations:** 1 Department of Anesthesiology, Netherlands Cancer Institute – Antoni van Leeuwenhoek, Plesmanlaan 121, Amsterdam, 1066 CX the Netherlands; 2 Department of Anesthesiology, Amsterdam UMC, Amsterdam, the Netherlands; 3 Department of Intensive Care Medicine, Amsterdam UMC, Amsterdam, the Netherlands; 4 Information Services, University Library, Vrije Universiteit Amsterdam, Amsterdam, the Netherlands; 5 Laboratory for Experimental Intensive Care and Anesthesiology (LEICA), Amsterdam UMC, Amsterdam, the Netherlands

## Abstract

**Objective:**

Arginine vasopressin (AVP) is a non-adrenergic vasopressor increasingly used in the management of shock. While it restores arterial pressure, its effects on the microcirculation remain uncertain, with potential for reduced regional perfusion and tissue oxygenation. The aim of this systematic review is to evaluate the effects of intravenous AVP and its analogues on microcirculatory perfusion across different haemodynamic conditions and vascular beds.

**Methods:**

A systematic search of MEDLINE, EMBASE, and Web of Science was conducted. Animal and human publications evaluating the effects of intravenous AVP or analogues on microcirculatory perfusion assessed with in vivo microcirculatory imaging or flowmetry techniques were included. Publications were grouped by haemodynamic condition and vascular bed.

**Results:**

Of 1693 records, 39 publications (31 animal, 7 human, 1 combined) were included, comprising 43 study-units and 152 individual measurements. Overall perfusion was decreased in 33%, unchanged in 48%, and increased in 19% of measurements, compared with baseline, saline, or other vasopressors. By haemodynamic condition, perfusion was decreased/unchanged/increased (n=study-units; % of measurements) in physiological conditions (*n* = 14: 57/38/4), vasodilatory or septic shock (*n* = 23: 24/55/21; versus baseline, fluid resuscitation or noradrenaline), haemorrhagic shock (*n* = 3: 17/17/67; versus baseline, saline or noradrenaline), cardiac dysfunction or arrest (*n* = 2: 0/67/33; versus adrenaline or noradrenaline) and cirrhosis-related portal hypertension (*n* = 1: 0/0/100). Across vascular beds: gastrointestinal tract (*n* = 18: 30/42/27), kidney (*n* = 9: medulla 77/23/0, cortex 27/64/9), sublingual bed (*n* = 7: 19/68/13), cerebral cortex (*n* = 2: 0/67/33), cutaneous bed (*n* = 6: 42/42/17), and conjunctiva (*n* = 1, 100/0/0).

**Conclusion:**

AVP and its analogues reduced microcirculatory perfusion most consistently under physiological conditions, whereas effects in vasodilatory or septic shock were more heterogeneous. Across vascular beds, the highest proportion of measurements showing reduced perfusion was in the renal medulla. These results highlight the divergent effects of vasopressin across vascular beds and clinical conditions.

**Supplementary Information:**

The online version contains supplementary material available at https://doi.org/10.1186/s40635-026-00983-5.

## Introduction

Vasopressors are the cornerstone in the management of shock. Noradrenaline (NE) is suggested by the Surviving Sepsis Campaign as first-line therapy due to its vasoconstrictive properties and wide availability. However, in patients with refractory shock, arginine vasopressin (AVP) is recommended as early adjunctive vasopressor rather than further increasing NE dosage [1]. AVP exerts vasoconstrictive effects through a catecholamine-independent mechanism, therefore reducing catecholamine requirements [2], and unlike NE, does not exert immunomodulatory effects [3, 4].

AVP exerts its effects through binding V1a, V1b, and V2 receptors, also designated V1, V3, and V2 respectively. V1a receptors, located on vascular smooth muscle in arteries and arterioles, mediate vasoconstriction. V1b receptors, on the anterior pituitary, affect the cortisol and androgen production. V2 receptors are expressed in renal distal tubules and collecting ducts, where they induce antidiuretic effects [5, 6]. Several analogues of vasopressin act on these same receptors, and are therefore used in place of AVP; these include terlipressin (TP), desmopressin (DDAVP), and selepressin [7].

Through V1a-receptor activation, vasopressin causes arteriolar vasoconstriction, increasing upstream resistance and potentially reducing downstream perfusion [8]. Moreover, V1a receptor expression varies between organs, being abundant in tissues such as the liver and kidney [9]. This heterogeneous distribution may underlie differing vasoconstrictive responses, and therefore differing effects on microcirculatory perfusion, across vascular beds. Importantly, while vasopressors are administered to restore haemodynamic stability, adequate macrocirculatory parameters do not necessarily reflect adequate microcirculatory perfusion [10]. This lack of haemodynamic coherence has been observed in septic patients and in patients after cardiac surgery with cardiopulmonary bypass (CPB) [11, 12].

AVP combined with catecholamines is associated with a lower incidence of atrial fibrillation (AF) and other arrhythmias [13, 14], and with a reduced incidence of post-operative acute kidney injury (AKI) compared with NE [15]. Unlike NE, AVP does not increase myocardial contractility [3]; this avoids a rise in myocardial oxygen demand, but provides no inotropic support against the accompanying increase in left ventricular afterload, which may be relevant where contractile reserve is limited. Potent vasoconstriction by vasopressin and its analogues can also reduce tissue perfusion and, in severe cases, cause organ ischaemia [16]. In individual patient data meta-analysis, digital ischaemia occurred more frequently with vasopressin, whereas mesenteric ischaemia and acute coronary syndrome events were similar between groups [14]. A pharmacovigilance analysis has nonetheless linked vasopressors to acute mesenteric ischaemia [17]. Whether these clinical signals reflect changes at the microvascular level is unknown, as such endpoints do not capture microcirculatory perfusion. Therefore, it is important to understand the mechanisms by which vasopressin affects the microcirculation.

Given the possible benefit of AVP over NE in less severe septic shock [18] and its increasing clinical use [19], we systematically reviewed the effect of vasopressin on microcirculatory perfusion across various clinical conditions and in various vascular beds.

## Methods

### Study design & registration

This systematic review was conducted and reported in accordance with the PRISMA 2020 statement [20]; the completed PRISMA 2020 checklist is provided in additional file (AF) 1. The protocol was prospectively registered with PROSPERO [21] (registration number: CRD420251051893) (AF 2)).

### Search strategy and screening

A systematic search for the influence of intravenous AVP or its analogues on microcirculatory perfusion in animals and humans was conducted in MEDLINE, Embase and Web of Science in consultation with a medical librarian (AVI) (AF 3). No date restriction was applied. The initial search was performed on April 11, 2025, and updated on March 19, 2026. After removal of duplicates, titles and abstracts were independently screened by two reviewers (JvZ, JK) using predefined criteria. Potentially relevant publications underwent full-text assessment. Disagreements were resolved through consultation of a third reviewer (CEB).

### Study selection

Eligibility criteria were specified according to the PECOS framework, in line with PRISMA 2020 recommendations [20]. We included randomised and non-randomised experimental and observational studies in animals of any species and humans of any age, whether healthy or with disease states including shock of any aetiology, exposed to intravenous AVP or an AVP analogue (terlipressin, selepressin, desmopressin, or a selective V1a-receptor agonist) given as a bolus or continuous infusion, compared with placebo, sham treatment, saline, another vasoactive agent, or within-subject baseline measurements obtained before administration, in which microcirculatory perfusion was assessed in vivo by sidestream dark-field imaging, incident dark-field imaging, orthogonal polarisation spectral imaging, light-guide probe, laser Doppler flowmetry, or intravital microscopy.

Reviews, case reports and research protocols were excluded, as were studies of topical administration and in vitro, ex vivo or in silico experiments. We also excluded studies reporting only tissue oxygenation, histological or biochemical outcomes, or vessel diameter, and studies measuring flow with fluorescein isothiocyanate dextran or microsphere techniques, as these do not provide direct, dynamic measures of microcirculatory flow.

### Risk of bias

Risk of bias was independently assessed by two reviewers (JvZ, JGK) and a third reviewer (CEB) in case of disagreements using study-design-specific tools.

*SYRCLE risk of bias tool* for in vivo animal studies [22]. For descriptive purposes, each domain was scored 0 (low), 1 (unclear), or 2 (high risk).

*Cochrane Risk of Bias (ROB) 2* for randomised controlled trials [23], with judgments categorised as low risk, some concerns, or high risk.

*ROBINS-I* for prospective non-randomised human publications [24], based on the microvascular flow index (MFI) outcome, and classified as low, moderate, serious, or critical risk of bias, as for RoB 2.

*Newcastle-Ottawa Scale (NOS) for t*he methodological quality of non-randomised publications [25]. Studies were scored across selection (0–4), comparability (0–2) and outcome (0–3) domains to a maximum of nine stars, with higher scores indicating lower risk of bias.

### Data extraction

Data were extracted independently by two reviewers (JvZ, JGK) and included publication, population, and intervention characteristics comprising AVP dose, mode and duration of administration and comparator agents). Microcirculatory perfusion was assessed through videomicroscopy-derived measures of capillary density and flow and laser Doppler–derived flux; full parameter definitions are provided in AF 4.

Throughout this review, ‘publication’ refers to a unique included paper, and ‘study-unit’ to an individual analysis within a publication. Each study-unit comprises one or more individual measurements, defined as a single reported microcirculatory perfusion parameter. For each study-unit, the overall direction of effect was classified as decreased, unchanged, or increased when all reported microcirculatory perfusion parameters changed in the same direction. Study-units in which parameters changed in more than one direction were classified as mixed. A heterogeneity-index change in the opposite direction to otherwise consistent flow and density parameters was not classified as mixed, since reduced heterogeneity reflects improved microvascular homogeneity.

### Statistical analysis

Due to high clinical and methodological heterogeneity among publications a quantitative meta-analysis was considered inappropriate. Publications were grouped according to study design (animal, human, or mixed), haemodynamic condition, and vascular bed. Publication bias was not tested statistically, given the expected small number of comparable publications.

To assess whether the direction of effect was related to methodological quality, a descriptive sensitivity analysis stratified by risk of bias was performed. As the SYRCLE tool does not define a threshold for overall risk of bias, animal study-units were dichotomised at median score. Human study-units were mapped onto a common two-level scale: RoB 2 low risk or some concerns, and ROBINS-I low or moderate risk, formed the lower stratum; ROBINS-I serious or critical risk, and a Newcastle-Ottawa score below five of nine, the higher stratum.

## Results

### Study selection

**Fig. 1:**
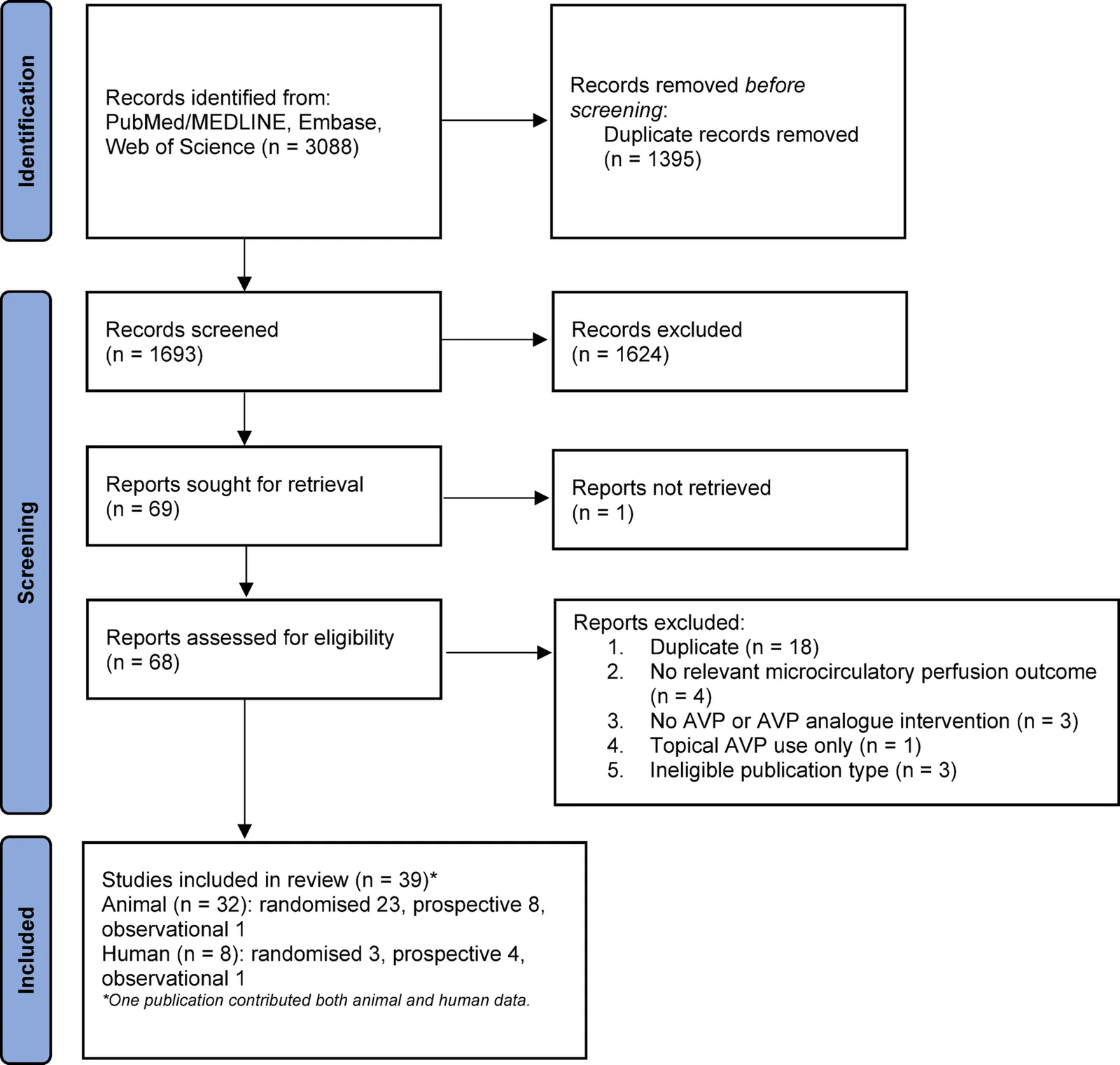
PRISMA 2020 flow diagram showing the study selection process for the systematic review of AVP and AVP analogues on microcirculatory outcomes following searches in MEDLINE, Embase, and Web of Science. The search was performed on 11 April 2025 and updated on 19 March 2026

The literature search yielded a total of 3088 records (Fig. 1). Following deduplication and title/abstract screening, full-texts of 68 publications were evaluated. Of these, 29 were excluded: duplicate publication (*n* = 18), no relevant microcirculatory perfusion outcomes (*n* = 4), no AVP or AVP analogue intervention (*n* = 3), topical AVP use only (*n* = 1), and ineligible publication type (*n* = 3).

### Study characteristics

In total 39 publications were included, animal (*n* = 31), human (*n* = 7), and combined (*n* = 1), ranging from 1983 to 2026. Among animal publications, 23 were randomised and 8 prospective; of the 7 human publications, 3 were randomised and 4 prospective. The combined publication was an observational cohort study. Baseline characteristics are given in Tables 1 and 2, and baseline fluid and AVP duration in AF 5 and 6.

**Table 1:** Characteristics, administration regimen, background fluid therapy and microcirculatory perfusion outcomes of the 35 animal study-units

Study [ref], species, design	Haemodynamic condition (model)	Intervention	Comparator	Background fluid therapy	Vascular bed and region	Measurements (*n*) and direction	Risk of bias
Correia 2001 [38]; Rabbit; Non-randomised in vivo animal study	Physiological (Normal state)	Selective V1a agonist; 30 ng/kg/min; 30 min; *n* = 7	saline (Isotonic saline: 50 µl/kg/min); *n* = 7	Hartmann’s solution 0.18 mL/kg/min + polygeline (4:1)	Renal; Kidney	2; Mixed	SYRCLE 5/20
Cowley 1998 [39]; Rat; Non-randomised in vivo animal study	Physiological (Normal state)	AVP and selective V1a agonist; 2 ng/kg/min; 5 days	baseline	Continuous saline carrier with AVP or V1a agonist (8.3 uL/min)	Renal; Kidney	4; Mixed	SYRCLE 5/20
Evans 2000 [40]; Rabbit; Non-randomised repeated-measures in vivo animal study	Physiological (Conscious rabbits)	Selective V1a agonist; 30 ng/kg/min; 20 min; *n* = 12	baseline (Isotonic saline: 50 µl/kg/min)	Saline 50 uL/kg/min for 20 min	Renal; Kidney	2; Mixed	SYRCLE 10/20
Franchini 1996 [41]; Rat; Non-randomised in vivo animal study	Physiological (Physiological (acute decerebration model))	AVP; 0.065–0.50 ng/kg/min; 30 min per dose; *n* = 4	saline; *n* = 3	Albumin colloid 2 mL/100 g/h; blood loss replaced with donor blood	Renal; Kidney	4; Mixed	SYRCLE 11/20
Franchini 1997 [42]; Rat; Non-randomised in vivo animal study	Physiological (Physiological (acute decerebration model))	AVP; 0.065–0.5 ng/kg/min; 30 min; *n* = 7	baseline	Albumin colloid 2 mL/100 g/h	Renal; Kidney	2; Mixed	SYRCLE 10/20
Friesenecker 2004 [37]; Hamster; RCT	Physiological (Normal state)	AVP; 0.0001 IU/kg/min; 30 min; *n* = 6	control; *n* = 7	Not reported	Cutaneous; Skin - dorsal skin	3; Decreased	SYRCLE 3/20
Friesenecker 2006 [43]; Hamster; RCT	Physiological (Normal state)	AVP; 0.0001 IU/kg/min; >=30 min; *n* = 6	NE (2 µg/kg/min); *n* = 5	Not reported	Cutaneous; Skin - dorsal skin	4; Mixed	SYRCLE 5/20
Holliger 1983 [34]; Rat; Non-randomised in vivo animal study	Physiological (Normal state)	AVP; bolus 1, 10, or 100 mU/kg & 14.3 mU/kg/min continuous; 3 boluses and 10 min continuous; *n* = 8	saline (Isotonic saline: 0.15 ml bolus followed by 0.03 ml/min); *n* = 5; 6; 10	Not reported	Gastrointestinal; Small intestine – villi	2; Decreased	SYRCLE 11/20
Kildal 2015 [36]; Pig; RCT	Physiological (Normal | Acute ischemic heart failure)	AVP; 0.001–0.012 IU/kg/min; First administration 20 min; repeated dosing, duration not reported; *n* = 8	AVP pre-ischemic; saline	Saline 10 mL/kg/h	Sublingual; Oral cavity- sublingual	5; Decreased	SYRCLE 8/20
Knotzer 2005 [35]; Pig; RCT	Physiological (Normal state)	AVP; 0.007–0.229 IU/kg/h; Incremental dosing at 20 min intervals; measurements up to 120 min; *n* = 8	saline; *n* = 8	Continuous Ringer lactate and gelatin to keep CVP 10–12 mmHg	Gastrointestinal; Small intestine - jejunal mucosa	1; Decreased	SYRCLE 6/20
Saeki 1991 (animals) [33]; Rat; Observational study	Physiological (Normal state)	AVP; 0.4 U/min; Rats 20 min; humans 10 min; *n* = 24	baseline	Not reported	Gastrointestinal; Stomach - gastric mucosa	1; Decreased	SYRCLE 13/20
Truse 2019 [44] — gastrointestinal unit; Dog; Randomised, blinded crossover	Physiological (Normal state)	AVP; 0.001–1.0 ng/kg/min; Continuous; dose increased every 30 min, measurement 30 min after each increase; *n* = 6	saline (Isotonic saline: 20 ml bolus ◊ 1 ml/kg/h for 2 h)	No baseline infusion	Gastrointestinal; Oral cavity | Stomach - mucosa	5; Mixed	SYRCLE 2/20
Truse 2019 [44] — cutaneous unit; Dog; Randomised, blinded crossover	Physiological (Normal state)	AVP; 0.001–1.0 ng/kg/min; Continuous; dose increased every 30 min, measurement 30 min after each increase; *n* = 6	saline (Isotonic saline: 20 ml bolus ◊ 1 ml/kg/h for 2 h)	No baseline infusion	Cutaneous; Oral cavity | Stomach - mucosa	11; Mixed	SYRCLE 2/20
Albert 2004 [59]; Rabbit; RCT	Vasodilatory/septic shock (Endotoxic shock)	AVP; 1–1000 ng; Incremental boluses at 5–10 min intervals; *n* = 6	baseline no-sepsis; baseline sepsis; *n* = 9	Saline + bicarbonate 4 mL/kg/h	Renal; Kidney	4; Mixed	SYRCLE 6/20
Asfar 2003 [53]; Rat; RCT	Vasodilatory/septic shock (Endotoxic shock)	Terlipressin + Gelofusine bolus (one arm); TP: 6 µg/kg bolus; TP boluses, repeated every 30 min if MAP < 90 mmHg; total duration not reported; *n* = 7; 18	LPS; LPS+fluid (Saline: 10 mL/kg/h); *n* = 17; 18; 18	Saline 10 mL/kg/h	Gastrointestinal; Small intestine - ileal serosa	2; Mixed	SYRCLE 3/20
Bomberg 2013 [49]; Pig; RCT	Vasodilatory/septic shock (Cardiopulmonary bypass)	AVP; To maintain baseline arterial pressure; 50 min; *n* = 6	baseline	Full electrolyte solution 10 mL/kg/h; blood loss replaced by gelatin	Gastrointestinal; Small intestine- jejunal serosa	2; Unchanged	SYRCLE 5/20
Bomberg 2014 [45]; Pig; RCT	Vasodilatory/septic shock (Cardiopulmonary bypass)	AVP; To maintain baseline arterial pressure; 50 min; *n* = 6	baseline	Full electrolyte solution 10 mL/kg/h; blood loss replaced by gelatin	Gastrointestinal; Colon -rectosigmoidal mucosa	1; Decreased	SYRCLE 4/20
Faivre 2005 [46]; Rabbit; Non-randomised in vivo animal study	Vasodilatory/septic shock (Endotoxemia)	AVP; 0.005 U/kg/min; 90 min; *n* = 7; 5	baseline (/uur)	Saline 20 mL bolus over 5 min	Renal; Kidney	2; Decreased	SYRCLE 7/20
Hiltebrand 2007 [52]; Pig; RCT	Vasodilatory/septic shock (Septic shock + non septic controls)	AVP; 0.06 U/kg/h; 180 min; *n* = 8; 8	crystalloids no-sepsis; crystalloids sepsis; *n* = 8; 8	Non-septic: Ringer lactate 20 mL/kg/h. Septic: gelatin 15 mL/kg over 45 min, then Ringer lactate 20 mL/kg/h	Gastrointestinal; Gastrointestinal tract - stomach, jejunum, colon mucosa and muscularis	8; Mixed	SYRCLE 5/20
Jufar 2026 [51] — cerebral unit; Sheep; RCT	Vasodilatory/septic shock (Cardiopulmonary bypass)	AVP; mean dose 0.017 IU/kg/min; Continuous, titrated to MAP 75–85 mmHg; duration not reported	NE; baseline CPB; metaraminol; phenylephrine (NE: 0.20 µg/kg/min | Phenylephrine: 2.0 µg/kg/min | Metaraminol: 2.0 µg/kg/min)	Continuous sodium lactate 2 mL/kg/h	Cerebral; Kidney and brain	4; Unchanged	SYRCLE 10/20
Jufar 2026 [51] — renal unit; Sheep; RCT	Vasodilatory/septic shock (Cardiopulmonary bypass)	AVP; mean dose 0.017 IU/kg/min; Continuous, titrated to MAP 75–85 mmHg; duration not reported	pre-infusion (NE: 0.20 µg/kg/min | Phenylephrine: 2.0 µg/kg/min | Metaraminol: 2.0 µg/kg/min)	Continuous sodium lactate 2 mL/kg/h	Renal; Kidney and brain	2; Unchanged	SYRCLE 10/20
Kopel 2008 [56]; Rabbit; Non-randomised in vivo animal study	Vasodilatory/septic shock (Endotoxic shock)	AVP; 1–1000 ng bolus; Boluses at 5–10 min intervals; dose-response experiment 2 h; *n* = 6	AVP no-endotox; *n* = 5	No baseline infusion	Gastrointestinal; Liver and gastrointestinal tract	3; Mixed	SYRCLE 8/20
Krejci 2007 [47] — gastrointestinal unit; Pig; RCT	Vasodilatory/septic shock	AVP; 0.06 IU/kg/h; 180 min; *n* = 8; 8	baseline no-sepsis; baseline sepsis (Ringer lactate: 20 mL/kg/h); *n* = 8; 8	Ringer lactate 15–20 mL/kg/h	Gastrointestinal; Liver, kidney, pancreas	4; Mixed	SYRCLE 5/20
Krejci 2007 [47] — renal unit; Pig; RCT	Vasodilatory/septic shock	AVP; 0.06 IU/kg/h; 180 min; *n* = 8; 8	Ringer no-sepsis; Ringer sepsis (Ringer lactate: 20 mL/kg/h); *n* = 8; 8	Ringer lactate 15–20 mL/kg/h	Renal; Liver, kidney, pancreas	2; Decreased	SYRCLE 5/20
Marcus 2026 [55]; Rat; RCT	Vasodilatory/septic shock (Colon ascendens stent peritonitis/sham surgery)	AVP + Levosimendan or milrinone; AVP: 13.5 mU/kg/h | Levosimendan: 12 µg/kg/10 min + 12 µg/kg/h | Milrinone: 50 µg/kg/10 min + 45 µg/kg/h; 90 min; *n* = 12; 12; 9; 9	baseline no-sepsis; baseline sepsis	Continuous crystalloid 2 mL/kg/h	Gastrointestinal; Liver, gastro-intestinal tract -serosa	8; Mixed	SYRCLE 4/20
Nakajima 2006 [57]; Rat; RCT	Vasodilatory/septic shock (Endotoxic shock)	AVP + L-arginine (one arm); To restore MAP to baseline; 60 min	AVP alone; saline	Continuous saline 0.3 mL/h	Gastrointestinal; Small intestine - villi mucosa	5; Mixed	SYRCLE 8/20
Qiu 2014 [58]; Rat; RCT	Vasodilatory/septic shock (Septic shock + non septic controls)	Terlipressin; 8 µg/kg/h; Continuous, to restore baseline MAP; duration not reported; *n* = 10	NE; saline (NE 4.0 µg/kg/min); *n* = 10; 5	NE + TP group: saline 5 mL/kg/h, extra saline if MAP fell > 40 mmHg over 30 min. Control: none	Gastrointestinal; Small intestine – Ileal mesentery	8; Mixed	SYRCLE 4/20
Schulz 2021 [54]; Rat; Randomised, blinded with repeated-measures dose escalation	Vasodilatory/septic shock (Mild and moderate sepsis)	AVP; 6.75–27 mU/kg/h; 30 min per dose, at least 90 min	baseline mild; baseline/vehicle mod; therapeutic AVP mod; vehicle mod	Ringer lactate 4 mL/h	Gastrointestinal; Colon - serosa Oral cavity -sublingual	10; Mixed	SYRCLE 2/20
Wafa 2015 [50]; Rat; RCT	Vasodilatory/septic shock (Endotoxemia (LPS)/non- endotoxemic control)	Desmopressin; 1 µg/kg bolus; Single bolus; *n* = 10; 10	LPS; saline; *n* = 10; 10	Not reported	Gastrointestinal; Small intestine – lumina mucosa	2; Unchanged	SYRCLE 3/20
Westphal 2004 [48]; Rat; RCT	Vasodilatory/septic shock	AVP; 0.006 U/min; Titrated: 15 +/- 5 min for + 20 mmHg, 30 +/- 5 min for + 40 mmHg; *n* = 8	saline; *n* = 7	Continuous 0.9% saline 2 mL/100 g/h	Gastrointestinal; Small intestine - Ileal mucosa	1; Decreased	SYRCLE 4/20
Cheung 2007 [60]; Sheep; RCT	Haemorrhagic shock	AVP; 0.4 IU/kg bolus + 4.8 IU/kg/h; 60 min; *n* = 3	pre-resusc	No reported	Conjunctival; Eye -conjunctival	1; Decreased	SYRCLE 6/20
González 2016 [61]; Piglet; RCT	Haemorrhagic shock	Terlipressin + Albumin + hypertonic saline; 20 µg/kg bolus; 30 min; *n* = 5	resusc treatments (Albumin + hypertonic saline: 15 ml/kg | Saline: 30 ml/kg); *n* = 6; 6	No baseline infusion	Sublingual; Oral cavity -sublingual	1; Unchanged	SYRCLE 5/20
Lima 2012 [62]; Hamster; RCT	Haemorrhagic shock	AVP + V1- or V2-receptor antagonist (two arms); AVP: 0.0001 U/kg/min | Anti-V1a: 1 µg/kg | Anti-V2: 1 µg/kg; 1 h; *n* = 10; 4; 4	NE post-resusc; saline resusc (NE: 2 µg/kg/min); *n* = 10; 10	No baseline infusion; resuscitation with saline bolus 2x lost blood volume	Cutaneous; Skin - dorsal skin	4; Increased	SYRCLE 6/20
Klein 2021 [63]; Pig; RCT	Cardiac dysfunction/arrest (ECMO + cardiac arrest)	AVP; 0.01–0.03 IU/min; Continuous to MAP 65 mmHg throughout 6 h experiment; *n* = 10	NE (0.2–2.0 µg/kg/min | Titrated to maintain a MAP at 65 mmHg); *n* = 10	Saline given if blood flow decreased	Sublingual; Oral cavity -sublingual	4; Unchanged	SYRCLE 8/20
Ristagno 2007 [64]; Pig; RCT	Cardiac dysfunction/arrest (Cardiac arrest + CPR)	AVP; 0.4 U/kg bolus; Single bolus; *n* = 5	epinephrine (30 µg/kg bolus); *n* = 5	Not reported	Cerebral; Brain	2; Increased	SYRCLE 4/20

**Fig. 2:**
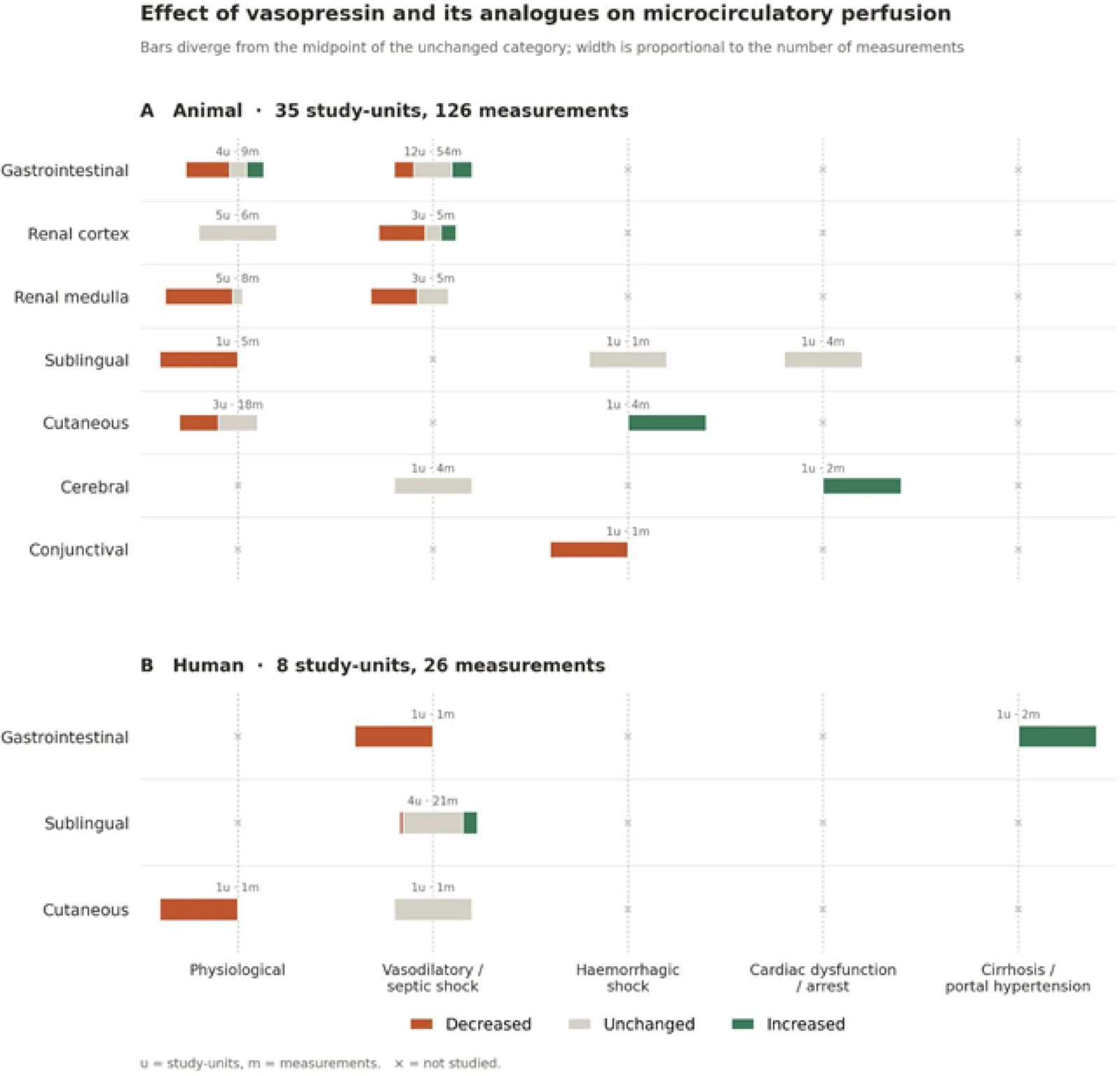
Direction of change in microcirculatory perfusion, by vascular bed and haemodynamic condition. All 152 measurements from the 43 included study-units are shown, separately for animal (panel **A**) and human (panel** B**) experiments. Rows represent vascular beds and columns represent haemodynamic conditions; shaded background panels distinguish the conditions, using the same colour scheme as in Tables 1 and 2. Bars diverge from the midpoint of the unchanged category, with bar width proportional to the number of measurements: decreased perfusion extends to the left (orange), increased perfusion to the right (green), and measurements showing no change are centred (grey). Each cell is labelled with the number of contributing study-units (u) and measurements (m); a cross denotes a combination that was not studied. The renal cortex and renal medulla are displayed as separate rows because the direction of effect differed between them; study-unit counts in these two rows are therefore not additive, as study-units that measured both compartments appear in each row, and the kidney contributed 9 unique study-units in total. One publication reported both animal and human data and contributes to both panels. Study-level detail for the individual study-units is given in Tables 1 and 2, and measurement-level detail in Additional file 8

Rows are grouped by haemodynamic condition, in the orderphysiological, vasodilatory or septic shock, haemorrhagic shock, cardiac dysfunction or arrest, and cirrhosis-related portal hypertension; the same order and condition labels are used in Fig. 2. One row per study-unit; publications contributing more than one study-unit appear more than once and are labelled by the vascular bed assessed. Renal cortex and medulla are reported as measurement locations within the renal vascular bed. Outcome parameters and measurement-level data are provided in additional file 8. Risk of bias is the overall judgement or score from Table 3; SYRCLE scores range from 0 to 20, with lower scores indicating lower risk of bias. AVP, arginine vasopressin; CPB, cardiopulmonary bypass; CPR, cardiopulmonary resuscitation; CVP, central venous pressure; ECMO, extracorporeal membrane oxygenation; IU, international units; LPS, lipopolysaccharide; MAP, mean arterial pressure; NE, noradrenaline; RCT, randomised controlled trial; SYRCLE, Systematic Review Centre for Laboratory animal Experimentation; TP, terlipressin

**Table 2:** Characteristics, administration regimen, background fluid therapy and microcirculatory perfusion outcomes of the 8 human study-units

Study [ref], species, design	Haemodynamic condition (model)	Intervention	Comparator	Background fluid therapy	Vascular bed and region	Measurements (*n*) and direction	Risk of bias
Wiles 1986 [29]; Human; Prospective study	Physiological (Normal state)	AVP; 2 U/h; 90 min; *n* = 8	saline	Saline as carrier (12.5 mL pre- and post-infusion)	Cutaneous; Hand, interdigital skinfold	1; Decreased	Moderate
Luckner 2006 [27]; Human; RCT	Vasodilatory/septic shock (Vasodilatory shock)	AVP + Noradrenaline; 4 IU/h; Not reported; *n* = 10	NE (NE: titrated to MAP ≥ 65 mmHg); *n* = 8	Colloid until stroke volume index no longer increased with volume loading	Cutaneous; Skin - forearm skin	1; Unchanged	Some concerns
Morelli 2011 (Crit Care) [28] — sublingual unit; Human; RCT	Vasodilatory/septic shock (Norepinephrine –dependent septic shock)	AVP and terlipressin + Noradrenaline; AVP: 0.04 IU/min | TP: 1 µg/kg/h; 6 h; *n* = 20; 20	NE (NE: titrated to MAP ≥ 65 mmHg); *n* = 20	6% hydroxyethyl starch 130/0.4 to maintain PAOP and CVP at baseline +/- 3 mmHg	Sublingual; Oral cavity - sublingual	10; Unchanged	Some concerns
Morelli 2011 (Intensive Care Med) [32] — sublingual unit; Human; Prospective study	Vasodilatory/septic shock	Terlipressin + Noradrenaline + colloids; 0.5 mg bolus; Single bolus; *n* = 20	baseline (NE + colloids: titrated to MAP ≥ 65)	6% hydroxyethyl starch 130/0.4 to maintain PAOP 8–12 and CVP 12–15 mmHg	Sublingual; Oral cavity - sublingual	5; Increased	Serious
Nascente 2017 [30]; Human; Prospective study	Vasodilatory/septic shock (Early septic shock (< 48 h vasopressor use))	AVP + Noradrenaline; 0.04 IU/min; 60 min; *n* = 18	NE (NE: titrated to MAP ≥ 65 mmHg)	Ringer lactate boluses to pulse pressure variation < = 13%	Sublingual; Oral cavity - sublingual	4; Unchanged	Serious
Nygren 2009 [31]; Human; Prospective study	Vasodilatory/septic shock (Catecholamine dependent vasodilatory shock)	AVP + Noradrenaline; 1.2–4.8 IU/h; 30 min per dose; *n* = 8	NE (NE: titrated to MAP ≥ 65)	Additional fluid only to replace urine loss	Gastrointestinal; Small intestine – Jejunal mucosa	1; Decreased	Serious
van Loon 2020 [26]; Human; RCT	Vasodilatory/septic shock (Systemic inflammation induced by LPS)	AVP; 0.05 IU/min; 5 h; *n* = 10	saline (NE: 0.05 µg/kg/min | Phenylephrine: 0.5 µg/kg/min); *n* = 10; 10; 10	Crystalloid 150 mL/h until 6 h after LPS, then 75 mL/h for 2 h	Sublingual; Oral cavity - sublingual	2; Unchanged	Some concerns
Saeki 1991 (humans) [33]; Human; Observational study	Cirrhosis-related portal hypertension	AVP; 0.4 IU/min; Rats 20 min; humans 10 min; *n* = 24	baseline	Not reported	Gastrointestinal; Stomach - gastric mucosa	2; Increased	4/9 stars

Rows are grouped by haemodynamic condition, in the order physiological, vasodilatory or septic shock, haemorrhagic shock, cardiac dysfunction or arrest, and cirrhosis-related portal hypertension; the same order and condition labels are used in Fig. 2. One row per study-unit; publications contributing more than one study-unit appear more than once and are labelled by the vascular bed assessed. Renal cortex and medulla are reported as measurement locations within the renal vascular bed. Outcome parameters and measurement-level data are provided in additional file 8. Risk of bias is the overall judgement or score from Table 3; AVP, arginine vasopressin; CVP, central venous pressure; IU, international units; LPS, lipopolysaccharide; MAP, mean arterial pressure; NE, noradrenaline; PAOP, pulmonary artery occlusion pressure; RCT, randomised controlled trial; TP, terlipressin

**Table 3:** Risk of bias assessment of the included publications

Study [ref]	Species	Design	Tool	Overall judgement or score	Main domains driving judgement
Albert 2004 [59]	Rabbit	RCT	SYRCLE (randomised)	6/20	See additional file 7, Table S7.1/S7.2
Asfar 2003 [53]	Rat	RCT	SYRCLE (randomised)	3/20	See additional file 7, Table S7.1/S7.2
Bomberg 2013 [49]	Pig	RCT	SYRCLE (randomised)	5/20	See additional file 7, Table S7.1/S7.2
Bomberg 2014 [45]	Pig	RCT	SYRCLE (randomised)	4/20	See additional file 7, Table S7.1/S7.2
Cheung 2007 [60]	Sheep	RCT	SYRCLE (randomised)	6/20	See additional file 7, Table S7.1/S7.2
Correia 2001 [38]	Rabbit	Non-randomised in vivo animal study	SYRCLE (non-randomised)	5/20	See additional file 7, Table S7.1/S7.2
Cowley 1998 [39]	Rat	Non-randomised in vivo animal study	SYRCLE (non-randomised)	5/20	See additional file 7, Table S7.1/S7.2
Evans 2000 [40]	Rabbit	Non-randomised repeated-measures in vivo animal study	SYRCLE (non-randomised)	10/20	See additional file 7, Table S7.1/S7.2
Faivre 2005 [46]	Rabbit	Non-randomised in vivo animal study	SYRCLE (non-randomised)	7/20	See additional file 7, Table S7.1/S7.2
Franchini 1996 [41]	Rat	Non-randomised in vivo animal study	SYRCLE (non-randomised)	11/20	See additional file 7, Table S7.1/S7.2
Franchini 1997 [42]	Rat	Non-randomised in vivo animal study	SYRCLE (non-randomised)	10/20	See additional file 7, Table S7.1/S7.2
Friesenecker 2004 [37]	Hamster	RCT	SYRCLE (randomised)	3/20	See additional file 7, Table S7.1/S7.2
Friesenecker 2006 [43]	Hamster	RCT	SYRCLE (randomised)	5/20	See additional file 7, Table S7.1/S7.2
González 2016 [61]	Piglet	RCT	SYRCLE (randomised)	5/20	See additional file 7, Table S7.1/S7.2
Hiltebrand 2007 [52]	Pig	RCT	SYRCLE (randomised)	5/20	See additional file 7, Table S7.1/S7.2
Holliger 1983 [34]	Rat	Non-randomised in vivo animal study	SYRCLE (non-randomised)	11/20	See additional file 7, Table S7.1/S7.2
Jufar 2026 [51]	Sheep	RCT	SYRCLE (randomised)	10/20	See additional file 7, Table S7.1/S7.2
Kildal 2015 [36]	Pig	RCT	SYRCLE (randomised)	8/20	See additional file 7, Table S7.1/S7.2
Klein 2021 [63]	Pig	RCT	SYRCLE (randomised)	8/20	See additional file 7, Table S7.1/S7.2
Knotzer 2005 [35]	Pig	RCT	SYRCLE (randomised)	6/20	See additional file 7, Table S7.1/S7.2
Kopel 2008 [56]	Rabbit	Non-randomised in vivo animal study	SYRCLE (non-randomised)	8/20	See additional file 7, Table S7.1/S7.2
Krejci 2007 [47]	Pig	RCT	SYRCLE (randomised)	5/20	See additional file 7, Table S7.1/S7.2
Lima 2012 [62]	Hamster	RCT	SYRCLE (randomised)	6/20	See additional file 7, Table S7.1/S7.2
Marcus 2026 [55]	Rat	RCT	SYRCLE (randomised)	4/20	See additional file 7, Table S7.1/S7.2
Nakajima 2006 [57]	Rat	RCT	SYRCLE (randomised)	8/20	See additional file 7, Table S7.1/S7.2
Qiu 2014 [58]	Rat	RCT	SYRCLE (randomised)	4/20	See additional file 7, Table S7.1/S7.2
Ristagno 2007 [64]	Pig	RCT	SYRCLE (randomised)	4/20	See additional file 7, Table S7.1/S7.2
Saeki 1991 (animals) [33]	Rat	Observational study	SYRCLE (non-randomised)	13/20	See additional file 7, Table S7.1/S7.2
Schulz 2021 [54]	Rat	Randomised, blinded with repeated-measures dose escalation	SYRCLE (randomised)	2/20	See additional file 7, Table S7.1/S7.2
Truse 2019 [44]	Dog	Randomised, blinded crossover	SYRCLE (randomised)	2/20	See additional file 7, Table S7.1/S7.2
Wafa 2015 [50]	Rat	RCT	SYRCLE (randomised)	3/20	See additional file 7, Table S7.1/S7.2
Westphal 2004 [48]	Rat	RCT	SYRCLE (randomised)	4/20	See additional file 7, Table S7.1/S7.2
Luckner 2006 [27]	Human	RCT	RoB 2	Some concerns	Deviations from intended interventions; selection of reported result; outcome measurement not assessed (MFI not reported)^1^
Morelli 2011 (Crit Care) [28]	Human	RCT	RoB 2	Some concerns	Randomisation; deviations from intended interventions
Morelli 2011 (Intensive Care Med) [32]	Human	Prospective study	ROBINS-I	Serious	Confounding
Nascente 2017 [30]	Human	Prospective study	ROBINS-I	Serious	Confounding; outcome measurement not assessed (MFI not reported)^1^
Nygren 2009 [31]	Human	Prospective study	ROBINS-I	Serious	Confounding; outcome measurement not assessed (MFI not reported)^1^
Saeki 1991 (humans) [33]	Human	Observational study	NOS	4/9 stars	Comparability 0 stars; outcome 1 star
van Loon 2020 [26]	Human	RCT	RoB 2	Some concerns	Randomisation; selection of reported result
Wiles 1986 [29]	Human	Prospective study	ROBINS-I	Moderate	Confounding; outcome measurement not assessed (MFI not reported)^1^

Domain-level assessments are provided in additional file 7. SYRCLE scores are summed across ten domains, each scored 0 (low risk), 1 (unclear) or 2 (high risk); the score therefore ranges from 0 to 20, with lower scores indicating lower risk of bias. RoB 2, ROBINS-I and NOS judgements are categorical and are not comparable with the SYRCLE score. ¹ NA, not assessed: the study did not report microvascular flow index, for which the outcome-measurement domain was evaluated. MFI, microvascular flow index; NOS, Newcastle-Ottawa Scale; RCT, randomised controlled trial; RoB 2, Cochrane Risk of Bias tool, version 2; ROBINS-I, Risk Of Bias In Non-randomised Studies of Interventions; SYRCLE, Systematic Review Centre for Laboratory animal Experimentation

AVP was administered in 36 study-units (29 animal, seven human), terlipressin in five (three animal, two human), desmopressin in one animal study-unit, and V1a-receptor agonists in three animal study-units. In animal studies, AVP was administered as a continuous weight-based infusion of 0.0001–0.017 U/kg/min in eight study-units; the remainder used weight-based infusions in other units, hourly infusions, fixed-rate or bolus regimens, or titration to a predefined haemodynamic target (additional file 6). Terlipressin was administered as a 6–20 µg/kg bolus or an 8 µg/kg/h infusion, desmopressin as a 1 µg/kg bolus, and a selective V1a-receptor agonist at 2–30 ng/kg/min. In human study-units, AVP was administered as continuous infusion of 0.02–0.4 U/min (*n* = 7). Terlipressin was administered in two studies as a 1 µg/kg/h infusion or a 0.5 mg bolus. Although selepressin was prespecified as an eligible intervention, no publication evaluating its effects on microcirculatory perfusion met the inclusion criteria.

### Risk of bias assessment

The three human randomised controlled trials [26–28] raised some concerns, mainly related to randomisation, deviations from intended interventions, and selective reporting. Of the four prospective human study-units [29–32] assessed with ROBINS-I, three [30–32] had an overall serious risk of bias, primarily due to confounding and participant selection, and one [29] was judged at moderate risk. The single observational human publication [33] scored four of nine stars on the Newcastle-Ottawa Scale. Four human study-units [27, 29–31] did not report MFI; because the outcome-measurement domain was assessed for MFI only, this domain was marked NA for those studies.

### Sensitivity analysis by risk of bias

Median SYRCLE score was five out of 20. Among animal measurements, perfusion was decreased, unchanged or increased in 35%, 46% and 19% of 80 measurements from lower-risk study-units, and in 41%, 41% and 17% of 46 measurements from higher-risk study-units. Among human study-units, perfusion was decreased, unchanged or increased in 7%, 93% and 0% of the 14 measurements from the lower-risk stratum, and in 17%, 33% and 50% of the 12 measurements from the higher-risk stratum. Results by haemodynamic condition and vascular bed are given in AF 7, Table S7.6-S7.8.

### Overall microcirculatory perfusion effects of vasopressin and analogues

Across the 39 included publications, microcirculatory perfusion was assessed with multiple imaging modalities, most commonly laser Doppler techniques (*n* = 16), intravital videomicroscopy (*n* = 9), SDF imaging (*n* = 7), IDF imaging (*n* = 3), OPS imaging (*n* = 1), and light-guide probe (*n* = 1); two publications used combined modalities (laser Doppler with microscopy).

Of the 43 study-units, 12 reported decreased microcirculatory perfusion across all assessed parameters, ten reported unchanged perfusion, four reported increased perfusion, and 17 reported mixed effects. Across all 152 perfusion measurements, 50 (33%), 73, (48%), and 29 (19%) showed decreased, unchanged, and increased perfusion, respectively.

### Microcirculatory assessment by haemodynamic condition in animal models

Results by haemodynamic condition are shown in Fig. 2; Tables 1 and 2, and AF 8.

#### Physiological conditions

Of 13 animal study-units, five reported an overall decrease in microcirculatory perfusion, two compared with saline and three with baseline [33–37]. A further five reported a divergent response within the same study-unit: unchanged cortical and decreased medullary perfusion compared with baseline or saline [38–42]. Two showed unchanged or decreased perfusion compared with control or NE, varying with dose and measurement location [43, 44]. In one, perfusion was unchanged or increased at 0.001–0.01 ng/kg/min and decreased at 1 ng/kg/min compared with baseline [44].

#### Vasodilatory or septic shock

Of 17 animal study-units, four reported decreased perfusion compared with baseline [45, 46], or with Ringer Lactate or saline [47, 48]. Four study-units in three publications reported unchanged microcirculatory perfusion compared with baseline or saline [49–51]. Two showed unchanged or decreased microcirculatory perfusion compared with baseline conditions or crystalloid resuscitation [47, 52], and one reported decreased perfusion compared with untreated endotoxaemic state, with restoration following a fluid challenge [53]. Four study-units showed unchanged or increased microcirculatory perfusion compared with baseline conditions, saline conditions, vehicle, AVP in non-endotoxic conditions, or AVP alone [54–57]. Two showed effects across all three directions, differing by comparator (saline vs. NE) [58] or by the presence of sepsis [59].

#### Haemorrhagic shock

Of three animal study-units, one showed decreased perfusion compared with pre-resuscitation phase [60], one unchanged perfusion compared with saline or albumin-based resuscitation [61], and one increased perfusion compared with saline resuscitation and NE during the post-resuscitation phase [62].

#### Cardiac dysfunction and arrest

One study-unit showed unchanged microcirculatory perfusion compared with NE [63] and one an increase compared with adrenaline [64].

### Microcirculatory assessment by haemodynamic condition in humans

Under physiological conditions, the single human study-unit reported decreased cutaneous perfusion compared with saline [29]. In vasodilatory or septic shock, six study-units were identified: four compared AVP or TP with NE against NE alone [27, 28, 30, 31], of which one reported decreased [31], and three unchanged perfusion [27, 28, 30]. One assessed AVP monotherapy against saline and reported unchanged perfusion [26], and one compared TP with NE against baseline and reported increased perfusion [32]. In cirrhosis-related portal hypertension, one study-unit reported increased gastric mucosal perfusion compared with baseline [33].

### Microcirculatory perfusion by vascular bed

The distribution of measurements across vascular beds and haemodynamic conditions is shown in Fig. 2; Tables 1 and 2; measurement-level data per study-unit are provided in AF 8.

In animal study-units, perfusion was decreased, unchanged or increased in 19, 28 and 16 of 63 gastrointestinal measurements, in three, seven and one of 11 renal cortical and in 10, three and zero of 13 renal medullary measurements, in five, five and zero of 10 sublingual measurements, in nine, nine and four of 22 cutaneous measurements, and in zero, four and two of six cerebral measurements. The single conjunctival measurement was decreased.

In human study-units, perfusion was decreased, unchanged or increased in one, zero and two of three gastrointestinal measurements, in one, 16 and four of 21 sublingual measurements, and in one, one and zero of two cutaneous measurements. No human study-unit assessed renal, cerebral or conjunctival microcirculation.

## Discussion

This systematic review of 39 publications found that effect of vasopressin and its analogues on microcirculatory perfusion varied with haemodynamic condition, vascular bed, and comparator: across 152 measurements, perfusion was decreased in 33%, and increased in 19%. These findings must be interpreted in context of three features of available evidence: predominance of animal data, concentration of human data in a single vascular bed, and short exposure throughout.

A key determinant of whether AVP affects microcirculatory perfusion appears to be the haemodynamic condition. Under physiological conditions, AVP tends to reduce microcirculatory perfusion, likely because increased vascular resistance limits capillary blood flow while macro- and microcirculatory remain coupled [10]. This effect was, however, more pronounced in study-units at higher risk of bias, in which 82% of measurements were decreased compared with 41% in study-units at lower risk. Results in healthy subjects are limited, as AVP is primarily used in critically ill patients [1]. In vasodilatory or septic shock the pattern becomes heterogeneous and no longer predominantly indicates deterioration, which may reflect the loss of vascular tone in vasodilatory states [65] and pre-existing microcirculatory disturbances. By restoring arteriolar tone, AVP increases precapillary resistance and perfusion pressure, which may improve flow in previously underperfused regions. These effects are inconsistent across studies, likely reflecting a loss of coherence between macro- and microcirculation [10]: arterial pressure increases, but the resulting microcirculatory response remains variable.

During cardiac dysfunction, microcirculatory responses to AVP were heterogeneous, consistent with a pathophysiology in which tissue perfusion is limited by reduced cardiac output and differs between vascular beds, depending on their susceptibility to hypoperfusion and regional autoregulation [66]. In haemorrhagic shock, improvement was largely confined to resuscitation phase, suggesting that restoration of circulating volume, rather than AVP itself, may drive it. Volume restoration alone, however, may not be sufficient: in experimental haemorrhagic shock, fluid resuscitation restored macrocirculatory variables while cremaster microcirculatory perfusion remained impaired, and renal cortical perfusion recovered only partially [67]. Improvement during resuscitation therefore cannot be attributed to volume replacement alone. Both conditions rest on few study-units and permit no firm conclusion. In bleeding trauma patients, low-dose vasopressin reduced blood product requirements [68]; as replacement in this setting is early and rapid, the benefit may again depend on concurrent volume restoration rather than a direct microcirculatory effect.

Although the haemodynamic condition shapes the overall response, variability within each condition suggests effects differ across vascular beds. Previous experimental [69] and retrospective clinical work has reported reductions in regional and microcirculatory intestinal blood flow after AVP [70]. In our data, gastrointestinal measurements showed no dominant direction, as the most frequently studied bed, this heterogeneity may reflect regional variation between jejunum, ileum, stomach, colon and mesentery rather than a uniform response.

The highest proportion of measurements showing reduced perfusion was in the renal medulla. In one included publication, a V1a-receptor agonist reduced medullary blood flow by 30% without altering cortical perfusion, total renal blood flow, or the diameters of juxtamedullary afferent and efferent arterioles [38], indicating that vasoconstriction occurred downstream of the glomerulus. Several considerations temper this apparent predominance of medullary reductions. The kidney was not among the most frequently studied vascular beds, contributing 9 of 43 study-units and 24 of 152 measurements, and 8 of the 10 medullary reductions derived from study-units at higher risk of bias. All renal data came from acute animal experiments and no human study-unit assessed renal microcirculation. Three of ten medullary reductions were observed with a selective V1a-receptor agonist rather than AVP, and in the single study-unit comparing both, medullary perfusion fell with the agonist but not with AVP [39]. Selective V1a-receptor agonists lack the concurrent V2-receptor stimulation of AVP, which attenuates medullary vasoconstriction through nitric oxide release [39, 71]; findings obtained with such agonists may therefore overstate the medullary effect of AVP itself. Vasopressin has been reported to improve renal outcomes in patients, which appears difficult to reconcile with reduced medullary perfusion in animals. In a trial in vasodilatory shock after cardiac surgery, AVP was associated with less AKI and RRT than noradrenaline [15]. This apparent discrepancy largely resolves when cortex and medulla are considered separately. Creatinine and renal replacement therapy reflect glomerular filtration, primarily a cortical function, and therefore report on the compartment in which perfusion was mainly unchanged; they would not detect a reduction confined to the medulla. Reduced medullary blood flow forms part of the normal antidiuretic response of vasopressin and, therefore, reduced perfusion alone does not necessarily indicate medullary ischaemic injury. Vasopressin was moreover given alongside or instead of noradrenaline, so any renal benefit may partly reflect reduced catecholamine exposure. Medullary perfusion may therefore fall without a detectable effect on global renal function, but whether this represents injury cannot be determined from the available evidence.

The sublingual vascular bed is easily accessible and can be examined non-invasively at bedside. Impaired sublingual microcirculation has been independently associated with longer ICU stay and higher 30-day mortality in shock [72], and with non-survival in cardiogenic shock [73]. In our data, sublingual perfusion in human study-units remained largely unchanged, whereas renal perfusion was reduced in the majority of measurements. Two readings are possible. The sublingual bed may genuinely respond differently from the renal circulation, in which case sublingual monitoring alone would underestimate the effect of AVP elsewhere. Alternatively, the human sublingual measurements were analysed semiquantitatively, most commonly as the microvascular flow index, and the predominance of unchanged values may partly reflect the limited sensitivity of the techniques then available; automated analysis now permits observer-independent quantification of capillary density and red blood cell velocity [74], these explanations are not mutually exclusive, and neither can be resolved from the present data. Either way, sublingual perfusion should not be assumed to represent the microcirculation as a whole during vasopressin administration.

The conditions under which vasopressin was given may themselves have shaped the responses observed. Exposure was brief: in all but one study-unit AVP was given for minutes to hours, and no human study-unit exceeded six hours. Several animal protocols used doses exceeding, on a weight-adjusted basis, those in the human study-units and those used clinically, but two considerations qualify this comparison. Weight-adjusted doses are not directly transferable between species, so a dose that appears supratherapeutic per kilogram need not be so in effect. The clinical reference is also not fixed: trials in vasoplegic and catecholamine-resistant shock have titrated vasopressin to rates above 0.03 U/min [15, 70], and the human study-units here used 0.02 to 0.4 U/min. Dose in any case does not explain the direction of effect: in one study-unit medullary perfusion fell across all doses tested [41]. Background fluid therapy ranged from starch-containing colloids to balanced crystalloid or none, and the effect of fluid type on the microcirculation remains debated [75]. In one study an AVP analogue reduced intestinal microcirculatory perfusion under baseline conditions but improved it with a fluid challenge [53], suggesting that relative hypovolaemia may predispose to impairment that volume optimisation can attenuate. Differences in exposure, dose and volume status may therefore account for part of the variation between study-units, and together with variation in vascular bed, comparator, agent and outcome measure, they are why the human data could not be pooled: a meta-analysis restricted to human study-units would rest on at most three comparable estimates and convey more precision than the underlying data support.

This review has three main limitations. First, evidence was heterogeneous in species, model, agent, dose and duration, so quantitative synthesis was therefore not appropriate. This also shaped the analysis: the direction of effect was classified categorically, which treats marginal and substantial changes alike and does not weight measurements by precision or sample size, and publications contributing several study-units are represented more than once. Second, human evidence is confined in scope and design. Of 43 study-units and 152 measurements, 35 and 126 respectively derive from animal experiments. Twenty-one of 26 human measurements were sublingual, the bed in which perfusion changed least, and no human study-unit assessed renal, cerebral or conjunctival microcirculation; the beds in which vasopressin most consistently reduced perfusion have therefore never been examined in patients. Among human study-units at lower risk of bias, 13 of 14 measurements were unchanged, whereas both study-units reporting increased perfusion were at higher risk of bias and compared measurements with a within-subject baseline rather than with a concurrent comparator. Third, risk of bias in the animal studies was frequently unclear because of limited reporting, and the stratification threshold was derived from the included data rather than from a validated cut-off.

Each of these limitations has a counterpart. The same variation that prevented pooling is itself a finding: across five haemodynamic conditions and seven vascular beds, the direction of effect depended on the bed, the condition, volume status and the comparator, a pattern that a single pooled estimate would have concealed. The predominance of animal data is likewise not only a weakness. Experimental animals are uniformly ill, free of comorbidity and polypharmacy, and receive controlled doses, so the preclinical evidence is internally more homogeneous than the clinical evidence; it also reaches vascular beds such as the renal medulla and the cerebral cortex that cannot be assessed in patients, which is why these observations exist at all. Finally, risk of bias was assessed for every publication with the instrument appropriate to its design and carried into a stratified sensitivity analysis, so the strength of the evidence behind each observation is visible rather than reported separately.

In conclusion, effects of AVP on microcirculatory perfusion are context-dependent, shaped by the underlying haemodynamic condition, vascular bed studied, volume status and comparator used. Perfusion tended to fall under physiological conditions, whereas in vasodilatory or septic shock, its effects are more heterogeneous and may be neutral or even beneficial. These conclusions rest largely on animal experiments, are confined to few vascular beds, and derive from short exposures. Future research should prioritise standardised microcirculatory assessment in patients, particularly in vascular beds not yet examined clinically and over the durations for which vasopressin is administered.

## Supplementary Information

Supplementary Material 1

Supplementary Material 2

Supplementary Material 3

Supplementary Material 4

Supplementary Material 5

Supplementary Material 6

Supplementary Material 7

Supplementary Material 8

Supplementary Material 9

## Data Availability

The data extraction template and the dataset extracted from the included studies are available from the corresponding author on reasonable request. No analytic code was generated, as no quantitative synthesis was performed.
