# Supplementary material for: Effect of vasopressin and its analogues on microcirculatory perfusion: a systematic review
Source: Intensive Care Med Exp. 2026 Oct 10;14:132. doi: 10.1186/s40635-026-00983-5 (PMC13653856; doi:10.1186/s40635-026-00983-5)
Supplement: Supplementary file 1 [file 40635_2026_983_MOESM1_ESM.pdf]

NIHR | National Institute for Health and Care Research

PROSPERO
International prospective register of systematic reviews

The Effect of Vasopressin on Microcirculatory Flow: A Systematic Review Across Imaging Modalities (SDF, IDF, Doppler, and Confocal Microscopy)

Judith van Zwol, Hanna Kruijsse, Charissa van den Brom

Citation
Judith van Zwol, Hanna Kruijsse, Charissa van den Brom. The Effect of Vasopressin on Microcirculatory Flow: A Systematic Review Across Imaging Modalities (SDF, IDF, Doppler, and Confocal Microscopy). PROSPERO 2025 CRD420251051893. Available from https://www.crd.york.ac.uk/PROSPERO/view/CRD420251051893.

REVIEW TITLE AND BASIC DETAILS

Review title

The Effect of Vasopressin on Microcirculatory Flow: A Systematic Review Across Imaging Modalities (SDF, IDF, Doppler, and Confocal Microscopy)

Original language title

English

Review objectives

Research question:

What is the effect of intravenous vasopressin or its analogues (administered as a bolus or continuous infusion) on microcirculatory perfusion parameters in animals and humans, compared to placebo, no intervention, or other vasoactive agents?

P (Population): Preclinical (animal) and clinical (human) subjects, regardless of health status, including both healthy individuals and those with underlying (created) disease or critical illness.

E (Exposure): Vasopressin or a vasopressin analogue, administered as an intravenous bolus or continuous infusion

C (Comparison): No intervention, placebo, or another vasoactive agent (e.g., noradrenalin)

O (Outcome): Microcirculatory perfusion parameters, including:

• Total vessel density (TVD)

• Perfused vessel density (PVD)

• Proportion of perfused vessels (PPV)

• Microvascular flow index (MFI)

• Heterogeneity index

• Blood flow velocity

• Perfusion units

• Functional capillary density

Context and rationale

Vasopressin (VAP) and norepinephrine (NE) are commonly used vasopressors in septic patients on the Intensive Care Unit and after cardiothoracic procedures. Both VAP and NE play a crucial role in stabilizing hemodynamics by increasing mean arterial pressure (MAP) with the primary goal of maintaining adequate organ perfusion. As for the different mechanism of action, extensive research has been conducted on the comparison of VAP and NE in septic patients, with a particular focus on acute kidney injury. The VANISH trial (Gordon et al. 2016) showed a tendency for a higher incidence of digital ischemia in patients receiving VAP compared to those receiving NE. Additionally, a meta-analysis on the survival of septic patients treated with VAP together with NE compared to NE alone, observes a significantly higher incidence of digital ischemia in patients receiving VAP (Yao et al. 2020). While a different study (Fage N. et al 2023) shows a renoprotective effect of VAP. Due to the strong vasoconstrictive effects of VAP, it could compromise microcirculation. Previous studies regarding microcirculatory perfusion when using VAP all use different outcome parameters, which makes it difficult to study the effect of VAP on the microcirculation, let alone draw a widely accepted conclusion on the effect of VAP on the microcirculation. This review provides an overview of the literature on the effect of VAP on the microcirculatory perfusion assessed using SDF, IDF, Doppler, and confocal microscopy. It is hypothesized that VAP leads to a decrease in microcirculatory perfusion in animals and patients over time

Keywords

Vasopressin; Arginine vasopressin; Microcirculation; Sidestream dark field imaging SDF; Incident Dark Field IDF; Laser-Doppler flowmetry; Doppler flow

SEARCHING AND SCREENING

Searches

Medline, EMBASE, web of science.

Language: Dutch, English

Search dates: 04/03/2025 & 11/04/2025

Inclusion criteria:

Systemic vasopressine infusion (bolus or continuous) over time with- or without additive of other vasoactive agent

Use of handheld videocamera (SDF/IDF), laser doppler or in vivo confocal microscopy

Animal- and human studies

General exclusion criteria:

Reviews

Case reports

Research protocols

Specific exclusion criteria:

Use of topical vasopressin

Laser confocal scanning microscopy

Use of fluorescein isothiocyanate dextran injection or injection of colored microspheres to determine flow

Outcomes based exclusion criteria:

Vessel diameter, not defined in abstract how measured

Dissection diameter (ex vivo)

Histology parameters

Endothelin-1 expression in mucosa

Microvascular hemoglobin oxygen saturation

Study design

Both randomized and nonrandomized study types will be included.

Included

Randomized controlled trials

Non-randomized controlled trials

Prospective cohort studies

Retrospective cohort studies

Case-control studies

Cross-sectional studies (if they assess microcirculatory flow)

Experimental animal studies (if relevant and include microcirculatory flow measurements with SDF, IDF, Doppler, or confocal microscopy)

In vivo physiological studies with original data on microcirculatory flow

Observational studies with quantitative analysis of microcirculatory perfusion

Excluded

Reviews

Case reports

Research protocols

Link to search strategy

A full search strategy has been uploaded to PROSPERO. The PDF may be accessed through this link https://www.crd.york.ac.uk/PROSPEROFILES/d4b1b9ef3aff855cbda4aeffaca1e65c.pdf.

ELIGIBILITY CRITERIA

Human disease modelled

Various acute and chronic conditions associated with disturbed hemodynamics or impaired microcirculatory perfusion, including but not limited to septic shock, hemorrhagic shock, cardiogenic shock, vasodilatory hypotension, and perioperative states. The review also includes models involving healthy subjects, where vasopressin is administered to study its physiological effects.

Animals/Population

Included

Animals with all created disease where vasopressin is administered will be included. Also healthy animals where vasopressin (in bolus or continuous) is administered will be included

Excluded

Ex vivo, in vitro, and in silico studies will be excluded because they do not represent whole-organism physiology.

In contrast, human in vivo studies will be included alongside animal studies, as they provide clinically relevant data on microcirculatory perfusion under physiological or pathological conditions.

Intervention(s) or exposure(s)

Included

Exposure to a single vasopressin intravenous bolus or continuous intravenous infusion

Excluded

Eligible control groups will include:

Placebo-treated controls

Sham-treated controls

Untreated controls

Controls treated with other vasoactive agents (e.g., noradrenalin)

Controls treated with both vasopressin and other vasoactive agent at the same time

Saline-treated controls

These controls are included to allow comparison of vasopressin or its analogues against both inactive and active comparators. No specific control type will be excluded as long as it allows for valid assessment of the microcirculatory effects of vasopressin.

Comparator(s) or control(s)

This review includes both comparative and non-comparative studies

Included

- Other vasoactive agents (e.g. noradrenalin, norepinephrin, phenylephrine)

- Saline

- Placebo

- Also studies without any comparison will be included

Excluded
No comparator based exclusion

Other selection criteria or limitations applied

Inclusion criteria: original article not published in English or Dutch, all publication dates.
Exclusion criteria: none.

OUTCOMES TO BE ANALYSED

Outcome measure(s)

Included
Total vessel density, perfused vessel density, proportion of perfused vessels, microvascular flow index, heterogeneity index, blood flow velocity, perfusion units, functional capillary density

Excluded
Outcomes based on:

Vessel diameter, not defined in abstract how it was measured

Dissection diameter (ex vivo)

Histology parameters

Endothelin-1 expression in mucosa

Microvascular hemoglobin oxygen saturation

DATA COLLECTION PROCESS

Study selection and data extraction

Procedure for study selection
Two reviewers will independently screen title-, and abstract of articles that emerged from the search. The screening process will be supported by Rayyan (Rayyan System Inc.), an online tool designed for systematic review screening. The same two reviewers will also read all articles included after title- and abstract screening. Final inclusion of a study was based on full text. In case of disagreement on inclusion at each phase, it was tried to reach consensus through discussion. In case of ongoing disagreement, an independent third reviewer was consulted to obtain final decision.

Prioritise the exclusion criteria
1) Original article not published in English or Dutch

2) microcirculatory parameters

3) Research protocols

4) (Systematic/Narrative) reviews

5) Case reports

6) No use of OPS, SDF, IDF, doppler flow or confocal microscopy

7) Use of laser confocal scanning microscopy

Outcomes described as:

8) Histology parameters

9) Endothelin-1 expression in mucosa

10) Vessel diameter

11) Dissection diameter (ex vivo)

12) Microvascular hemoglobin oxygen saturation

13) In vitro studies

14) Ex vivo studies

15) In silico studies

Methods for data extraction
To provide an overview on the available data and to draw a conclusion, data expressed in the included studies will be abstracted and sorted by the same 2 reviewers (JvZ, HK). Data will be divided in baseline characteristics and information on intervention. Baseline characteristics include study design, study population, experimental set-up, measurement tools, and outcome measurements. Information on intervention will include: VAP therapy and dosage, duration of administration, main outcomes, and relevant additional data. If only graphs are displayed in studies, without the exact numbers needed to determine the parameter outcomes, a copy of the raw data will be requested from the corresponding author. If there are any discrepancies, a third reviewer will help to extract data (CvB).

Data to be extracted: study design
Study type: randomized controlled trial, non-randomized controlled trial, prospective cohort, retrospective cohort, case control, crossover trial, observational study, case series, experimental (preclinical) animal studies.

Inclusion period (in years)

Country of publication

Population size

Setting of study: e.g. ICU, perioperative, animal model

Number and nature of experimental groups: e.g. vasopressin vs placebo/control, or comparison with other vasoactive agents

Presence and type of control group(s)

Blinding: e.g. single-blind, double-blind, open-label

Patient characteristics & animal characteristics

In case of animal study: which animal

Data to be extracted: animal model
All animal models will be included. No exclusion based on specific characteristics. Various acute and chronic conditions associated with disturbed hemodynamics or impaired

microcirculatory perfusion, including but not limited to septic shock, hemorrhagic shock, cardiogenic shock, vasodilatory hypotension, and perioperative states. The review also includes models involving healthy animals, where vasopressin is administered to study its physiological effects.

Data to be extracted: intervention of interest
The intervention (vasopressin (VAP) administration) will be extracted as:

VAP:

- Bolus

- Continuous infusion. If yes: duration of infusion

- Dose/dosage of VAP

- VAP alone or in combination with other vasoactive agent. If yes: report of other vasoactive dose, duration and information on bolus or continuous infusion.

- Timing (e.g. which timepoint during ICU stay, perioperative or during disease) of microcirculatory assessment

Data to be extracted: primary outcome(s)
- Which technique was (SDF, IDF, OPS, doppler flow, confocal microscopy) used to obtain the primary outcome parameters.

- Microcirculatory parameters defining the microcirculatory flow (Total vessel density, perfused vessel density, proportion of perfused vessels, microvascular flow index, heterogeneity index, blood flow velocity, perfusion units, functional capillary density)

- Which vessel the microcirculatory parameters were derived from

Risk of bias and/or quality assessment
By use of SYRCLE's risk of bias tool

Method for risk of bias and/or quality assessment
Risk of bias and/or methodological quality of the included studies will be assessed independently by two reviewers. We will use tools depending on the study design:

For randomized controlled trials, the Cochrane Risk of Bias 2 tool will be used.

For non-randomized studies, we will use the ROBINS-I tool.

For observational studies such as cohort and case-control studies, the Newcastle-Ottawa Scale will be applied.

For experimental animal studies, we will use the SYRCLE Risk of Bias tool.

Each study will be assessed independently by two reviewers. Any discrepancies between reviewers will be discussed and resolved through consensus. If consensus cannot be reached, a third reviewer will be consulted. The results of the risk of bias assessments will be reported in tables.

PLANNED DATA SYNTHESIS

Strategy for data synthesis

Planned approach

We will first provide a narrative synthesis of all included studies, describing study characteristics, patient population, vasopressin- and other vasoactive agent used dosing, and methods used to assess the microcirculation (e.g. IDF, SDF, laser Doppler, in vivo confocal microscopy). For each outcome measure related to microcirculatory parameters (e.g. perfused vessel density, microvascular flow index etc.), we will evaluate whether a quantitative synthesis (meta-analysis) is appropriate.

We anticipate a high degree of variability between studies in terms of patient populations, vasopressin dosing, duration of administration, and microcirculatory assessment techniques. Due to this expected clinical and methodological heterogeneity, a quantitative synthesis (meta-analysis) is unlikely to be feasible.

Therefore, we plan to conduct a narrative synthesis for all outcome measures. This synthesis will summarize findings according to study design, patient population (e.g. ICU vs perioperative), vasopressin intervention characteristics (e.g. dose, timing), and the method used to assess the microcirculation. The goal is to identify consistent patterns or differences across studies, rather than to pool effect sizes.

Analysis of subgroups or subsets

Subgroup analyses
None planned

Sensitivity
Given the expected heterogeneity in the population studied (human/animal), vasopressin dosing, duration of administration, the additive of other vasoactive agents, and microcirculatory assessment techniques (IDF, SDF, laser Doppler, in vivo confocal microscopy), we anticipate on conducting a narrative synthesis rather than a meta-analysis. Therefore, sensitivity analyses will be primarily qualitative.

For each microcirculatory parameter we will assess whether findings are consistent when:

Conclusions change when excluding studies with high risk of bias (as assessed using RoB 2, ROBINS-I, NOS, or SYRCLE).

Whether findings differ between randomized controlled trials and observational or experimental studies.

Whether results are consistent when comparing studies using different measurement techniques (IDF, SDF, laser Doppler and in vivo confocal microscopy).

Whether studies using similar dosing and timing of vasopressin yield more comparable outcomes.

Although formal statistical sensitivity analyses may not be feasible due to limited data or high variability, these planned assessments will help us determine the robustness and reliability of our overall conclusions.

Publication bias
Given the expected heterogeneity in study designs, patient populations, and outcome measures, and the likely small number of comparable studies, a formal meta-analysis may not be feasible. Therefore, a quantitative assessment of publication bias (e.g. using funnel plots or Egger's test) is not planned at this stage.

In the context of a narrative synthesis, we will also consider the potential for publication bias by evaluating the presence of unpublished studies (through clinical trial registries).

REVIEW AFFILIATION, FUNDING AND PEER REVIEW

Review team members

Miss Judith van Zwol (review guarantor and contact) Amsterdam UMC. Netherlands.

No conflict of interest declared.

Miss Hanna Kruijsse. Amsterdam UMC. Netherlands.

No conflict of interest declared.

Dr Charissa van den Brom. Amsterdam UMC. Netherlands.

No conflict of interest declared.

Named contact

Miss Judith van Zwol (judith.vanzwol@amsterdamumc.nl). Amsterdam UMC. Netherlands.

Review affiliation

Netherlands Cancer Institute – Antoni van Leeuwenhoek (NKI-AVL)

Plesmanlaan 121, 1066 CX Amsterdam, The Netherlands

Funding source

Review has no specific/external funding but is supported by guarantor/review team (non-commercial) institutions.

TIMELINE OF THE REVIEW

Review timeline

Start date: 13 May 2025. End date: 31 December 2025.

Date of first submission to PROSPERO

15 May 2025

Date of registration in PROSPERO

27 May 2025

CURRENT REVIEW STAGE

Publication of review results

Results of the review will be published in English.

Stage of the review at this submission

Review stage	Started	Completed
Pilot work	✓	✓
Formal searching/study identification	✓	✓

Review stage	Started	Completed
Screening search results against inclusion criteria	✓	✓
Data extraction or receipt of IPD		
Risk of bias/quality assessment		
Data synthesis		

Review status
The review is currently planned or ongoing.

ADDITIONAL INFORMATION

Review type
Animal model review, Experimental animal exposure review, Pre-clinical animal intervention review

Additional information
Both human, and animals will be included as study subjects

PROSPERO version history

- Version 1.0, published 27 May 2025

Review conflict of interest
Declared individual interests are recorded under team member details.. No additional interests are recorded for this review.

Country
Netherlands

Medical Subject Headings
Vasopressins; Blood Flow Velocity; Microcirculation; Humans; Animals

Disclaimer
The content of this record displays the information provided by the review team. PROSPERO does not peer review registration records or endorse their content.

PROSPERO accepts and posts the information provided in good faith; responsibility for record content rests with the review team. The guarantor for this record has affirmed that the information provided is truthful and that they understand that deliberate provision of inaccurate information may be construed as scientific misconduct.

PROSPERO does not accept any liability for the content provided in this record or for its use. Readers use the information provided in this record at their own risk.

Any enquiries about the record should be referred to the named review contact
