# Supplementary material for: Effect of vasopressin and its analogues on microcirculatory perfusion: a systematic review
Source: Intensive Care Med Exp. 2026 Oct 10;14:132. doi: 10.1186/s40635-026-00983-5 (PMC13653856; doi:10.1186/s40635-026-00983-5)
Supplement: Supplementary file 2 [file 40635_2026_983_MOESM2_ESM.docx]

**Additional file 3: search strategy**

MEDLINE

| **Search** | **Query** | **Results** |
| --- | --- | --- |
| #3 | 1 and 2 | **845** |
| #2 | exp "Vasopressins"/ or ("vasopressin*" or (("Antidiuretic" or "Anti-diuretic") adj4 "hormone*") or "arginine-vasopressin" or "AVP" or "ADH" or "argipressin" or "beta-Hypophamine" or "beta Hypophamine" or "Pitressin" or "Vasostrict" or "terlipressin" or "desmopressin").ti,ab,kf. | 62551 |
| #1 | exp "Microcirculation"/ or ("microcirculat*" or "micro-circulat*" or "microvasc*" or "micro-vasc*" or "microvess*" or "micro-vess*" or "arteriol*" or "metarteriol*" or "meta-arteriol*" or "capillaries" or "venule*" or (("medullary" or "splanchnic") adj3 ("blood-flow" or "micro-circ*" or "microcirc*"))).ti,ab,kf. | 199260 |

Embase

| **Search** | **Query** | **Results** |
| --- | --- | --- |
| #5 | #3 NOT #4 | **1,155** |
| #4 | #3 AND ('conference abstract'/it OR 'conference paper'/it OR 'conference review'/it) | 228 |
| #3 | #1 AND #2 | 1,383 |
| #2 | 'vasopressin derivative'/exp OR ('vasopressin*':ti,ab,kw OR (('antidiuretic' OR 'anti-diuretic') NEAR/4 'hormone*'):ti,ab,kw OR 'arginine-vasopressin':ti,ab,kw OR 'avp':ti,ab,kw OR 'adh':ti,ab,kw OR 'argipressin':ti,ab,kw OR 'beta-hypophamine':ti,ab,kw OR 'beta hypophamine':ti,ab,kw OR 'pitressin':ti,ab,kw OR 'vasostrict':ti,ab,kw OR 'terlipressin':ti,ab,kw OR 'desmopressin':ti,ab,kw) | 101,951 |
| #1 | 'microcirculation'/exp OR ('microcirculat*':ti,ab,kw OR 'micro-circulat*':ti,ab,kw OR 'microvasc*':ti,ab,kw OR 'micro-vasc*':ti,ab,kw OR 'microvess*':ti,ab,kw OR 'micro-vess*':ti,ab,kw OR 'arteriol*':ti,ab,kw OR 'metarteriol*':ti,ab,kw OR 'meta-arteriol*':ti,ab,kw OR 'capillaries':ti,ab,kw OR 'venule*':ti,ab,kw OR (('medullary' OR 'splanchnic') NEAR/3 ('blood-flow' OR 'micro-circ*' OR 'microcirc*')):ti,ab,kw) | 272,134 |

Web of Science

| **Search** | **Query** | **Results** |
| --- | --- | --- |
| #3 | 1 and 2 | **1,043** |
| #2 | TS=("vasopressin*" or (("Antidiuretic" or "Anti-diuretic") NEAR/4 "hormone*") or "arginine-vasopressin" or "AVP" or "ADH" or "argipressin" or "beta-Hypophamine" or "beta Hypophamine" or "Pitressin" or "Vasostrict" or "terlipressin" or "desmopressin") | 74,795 |
| #1 | TS=("microcirculat*" or "micro-circulat*" or "microvasc*" or "micro-vasc*" or "microvess*" or "micro-vess*" or "arteriol*" or "metarteriol*" or "meta-arteriol*" or "capillaries" or "venule*" or (("medullary" or "splanchnic") NEAR/3 ("blood-flow" or "micro-circ*" or "microcirc*"))) | 230,170 |

Summary table

| **Database** | **References found** | **After Deduplication** |
| --- | --- | --- |
| MEDLINE | **845** |  |
| Embase | **1,155** |  |
| Web of Science | **1,043** |  |
| **Total** | **3,043** | **1680**  (1363 duplicates) |

Search update 19-03-2026

***MEDLINE***

| **Search** | **Query** | **Results** |
| --- | --- | --- |
| #3 | 1 and 2 | **858** |
| #2 | exp "Vasopressins"/ or ("vasopressin*" or (("Antidiuretic" or "Anti-diuretic") adj4 "hormone*") or "arginine-vasopressin" or "AVP" or "ADH" or "argipressin" or "beta-Hypophamine" or "beta Hypophamine" or "Pitressin" or "Vasostrict" or "terlipressin" or "desmopressin").ti,ab,kf. | 63783 |
| #1 | exp "Microcirculation"/ or ("microcirculat*" or "micro-circulat*" or "microvasc*" or "micro-vasc*" or "microvess*" or "micro-vess*" or "arteriol*" or "metarteriol*" or "meta-arteriol*" or "capillaries" or "venule*" or (("medullary" or "splanchnic") adj3 ("blood-flow" or "micro-circ*" or "microcirc*"))).ti,ab,kf. | 207264 |

***Embase***

| **Search** | **Query** | **Results** |
| --- | --- | --- |
| #5 | #3 NOT #4 | **1,175** |
| #4 | #3 AND ('conference abstract'/it OR 'conference paper'/it OR 'conference review'/it) | 228 |
| #3 | #1 AND #2 | 1,383 |
| #2 | 'vasopressin derivative'/exp OR ('vasopressin*':ti,ab,kw OR (('antidiuretic' OR 'anti-diuretic') NEAR/4 'hormone*'):ti,ab,kw OR 'arginine-vasopressin':ti,ab,kw OR 'avp':ti,ab,kw OR 'adh':ti,ab,kw OR 'argipressin':ti,ab,kw OR 'beta-hypophamine':ti,ab,kw OR 'beta hypophamine':ti,ab,kw OR 'pitressin':ti,ab,kw OR 'vasostrict':ti,ab,kw OR 'terlipressin':ti,ab,kw OR 'desmopressin':ti,ab,kw) | 101,951 |
| #1 | 'microcirculation'/exp OR ('microcirculat*':ti,ab,kw OR 'micro-circulat*':ti,ab,kw OR 'microvasc*':ti,ab,kw OR 'micro-vasc*':ti,ab,kw OR 'microvess*':ti,ab,kw OR 'micro-vess*':ti,ab,kw OR 'arteriol*':ti,ab,kw OR 'metarteriol*':ti,ab,kw OR 'meta-arteriol*':ti,ab,kw OR 'capillaries':ti,ab,kw OR 'venule*':ti,ab,kw OR (('medullary' OR 'splanchnic') NEAR/3 ('blood-flow' OR 'micro-circ*' OR 'microcirc*')):ti,ab,kw) | 272,134 |

***Web of Science***

| **Search** | **Query** | **Results** |
| --- | --- | --- |
| #3 | 1 and 2 | **1,055** |
| #2 | TS=("vasopressin*" or (("Antidiuretic" or "Anti-diuretic") NEAR/4 "hormone*") or "arginine-vasopressin" or "AVP" or "ADH" or "argipressin" or "beta-Hypophamine" or "beta Hypophamine" or "Pitressin" or "Vasostrict" or "terlipressin" or "desmopressin") | 76,664 |
| #1 | TS=("microcirculat*" or "micro-circulat*" or "microvasc*" or "micro-vasc*" or "microvess*" or "micro-vess*" or "arteriol*" or "metarteriol*" or "meta-arteriol*" or "capillaries" or "venule*" or (("medullary" or "splanchnic") NEAR/3 ("blood-flow" or "micro-circ*" or "microcirc*"))) | 239,637 |

***Summary table***

| **Database** | **References found** | **After Deduplication** | **New references**   **as of 19.03.2026** |
| --- | --- | --- | --- |
| MEDLINE | **858** |  |  |
| Embase | **1,175** |  |  |
| Web of Science | **1,055** |  |  |
| **Total** | **3,088** | **1693**   (1395 duplicates) | **27** |
