# Supplementary material for: Effect of vasopressin and its analogues on microcirculatory perfusion: a systematic review
Source: Intensive Care Med Exp. 2026 Oct 10;14:132. doi: 10.1186/s40635-026-00983-5 (PMC13653856; doi:10.1186/s40635-026-00983-5)
Supplement: Supplementary file 4 [file 40635_2026_983_MOESM4_ESM.docx]

**Additional file 5: baseline fluid infusion table**

| Study | Fluid infusion administered in all included subjects* |
| --- | --- |
| Albert et al., 2004 [59] | Saline + bicarbonate at 4 mL/kg/h |
| Asfar et al., 2003 [53] | Saline at 10 mL/kg/h. |
| Bomberg et al., 2013 [49] | Full electrolyte solution 10 mL/kg/h.  Intra-operative blood loss was replaced by gelatin solution |
| Bomberg et al., 2014 [45] | Full electrolyte solution 10 mL/kg/h.  Intra-operative blood loss was replaced by gelatin solution |
| Cheung et al., 2007 [60] | Not reported |
| Correia et al., 2001 [38] | Hartmann’s solution 0.18 ml/kg/min + polygeline (4:1) |
| Cowley et al., 1998 [39] | Continuous saline carrier with added AVP or V1 agonist (8.3 µL/min) |
| Evans et al., 2000 [40] | 50 µL/kg/min saline for 20 min |
| Faivre et al., 2005 [46] | 20 mL of saline in 5 min bolus |
| Franchini et al., 1996 [41] | Albumin colloid solution at 2 mL/100g/h  Blood losses replaced with donor blood |
| Franchini et al., 1997 [42] | Albumin colloid solution at 2 mL/100g/h |
| Friesenecker et al., 2004 [37] | Not reported |
| Friesenecker et al., 2006 [43] | Not reported |
| Gonzalez et al., 2016 [61] | No baseline infusion |
| Hiltebrand et al., 2007 [52] | Non septic group: Ringer Lactate 20 mL/kg/h  Septic group: 15 mL/kg gelatin resuscitation in 45 min bolus followed by continuous Ringer lactate 20 mL/kg/h |
| Holliger et al., 1983 [34] | Not reported |
| Jufar et al., 2026 [51] | Continuous sodium lactate 2 ml/kg/h |
| Kildal et al., 2015 [36] | Saline at 10 mL/kg/h |
| Klein et al., 2021 [63] | Saline was used in the event of decreased blood flow |
| Knotzer et al., 2005 [35] | Continuous Ringer Lactate solution and gelatine to keep CVP between 10 and 12 mmHg |
| Kopel et al., 2008 [56] | No baseline infusion |
| Krejci et al., 2007 [47] | Ringer Lactate infusion at 15-20 mL/kg/h |
| Lima et al., 2012 [62] | No baseline infusion  Resuscitation volume: saline bolus of 2x lost bloodvolume |
| Luckner et al., 2006 [27] | Colloid solutions until the stroke volume index could not be increased further by volume loading. |
| Marcus et al., 2026 [55] | Continuous crystalloid 2 ml/kg/h |
| Morelli et al., 2011 [28] | Fluid challenge of 6% hydroxyethyl starch 130/0.4, to maintain PAOP and CVP at baseline ± 3 mmHg. |
| Morelli et al., 2011 [32] | Fluid challenge of 6% hydroxyethyl starch 130/0.4 to maintain PAOP 8–12 mmHg and CVP 12–15 mmHg. |
| Nascente et al., 2017 [30] | Bolus Ringer Lactate to decrease pulse pressure variation ≤ 13, in case of unavailable PPV bolus were repeated until cardiac output increase was ≤ 10%. |
| Nakajima et al., 2006 [57] | Continuous saline at 0.3 mL/h |
| Nygren et al., 2009 [31] | Additional fluids only to replace urine loss |
| Qiu et al., 2014 [58] | NE + TP group: 5 ml/kg/h saline and in case of MAP dropping > 40 mmHg over 30 min extra fluid resuscitation saline.  Control group: no continuous fluid administration |
| Ristagno et al., 2007 [64] | Not reported |
| Saeki et al, 1991 [33] | Not reported |
| Schulz et al., 2021 [54] | Ringer Lactate at 4 mL/h |
| Truse et al., 2019 [44] | No baseline infusion |
| Van Loon et al., 2020 [26] | Crystalloid solution 150 mL/h until 6 h after LPS, then 75 mL/h for 2 h. |
| Wafa et al., 2015 [50] | Not described |
| Westphal et al., 2004 [48] | Continuous crystalloid solution (0.9% saline) 2 ml/100g/h |
| Wiles et al., 1986 [29] | Saline was used as carrier solution (12.5 mL pre- and post-infusion) for AVP. |

* Unless specific groups stated in this column
