# Supplementary material for: Effect of vasopressin and its analogues on microcirculatory perfusion: a systematic review
Source: Intensive Care Med Exp. 2026 Oct 10;14:132. doi: 10.1186/s40635-026-00983-5 (PMC13653856; doi:10.1186/s40635-026-00983-5)
Supplement: Supplementary file 5 [file 40635_2026_983_MOESM5_ESM.docx]

**Additional file 6: duration of infusion**

| Table S6: AVP/TP/DDAVP administration | |
| --- | --- |
| **Study** | **Duration** |
| Albert et al., 2004 [59] | Incremental AVP bolus with 5–10 min intervals |
| Asfar et al., 2003 [53] | TP boluses: first dose after fluid challenge, additional doses every 30 min if MAP <90 mmHg. Total duration not reported |
| Bomberg et al., 2013 [49] | 50 min |
| Bomberg et al., 2014 [45] | 50 min |
| Cheung et al., 2007 [60] | 60 min |
| Correia et al., 2001 [38] | 30 min |
| Cowley et al., 1998 [39] | 5 days |
| Evans et al., 2000 [40] | 20 min |
| Faivre et al., 2005 [46] | 90 min |
| Franchini et al., 1996 [41] | 30 min per dose |
| Franchini et al., 1997 [42] | 30 min |
| Friesenecker et al., 2004 [37] | 30 min |
| Friesenecker et al., 2006 [43] | ≥30 min |
| Gonzalez et al., 2016 [61] | 30 min |
| Hiltebrand et al., 2007 [52] | 180 min |
| Holliger et al., 1983 [34] | 3 bolus & 10 min continuous |
| Jufar et al., 2026 [51] | Continuous AVP infusion. Duration not reported; to achieve a MAP of 75–85 mmHg. |
| Kildal et al., 2015 [36] | First AVP administration 20 min; repeated continuous dosing after first administration: duration not reported. |
| Klein et al., 2021 [63] | Continuous IV AVP infusion (MAP target 65 mmHg), duration not reported but administered throughout 6 h experiment. |
| Knotzer et al., 2005 [35] | Incremental AVP dosing at 20-min intervals, measurements continued up until 120 min |
| Kopel et al., 2008 [56] | AVP boluses with 5–10 min intervals. Total dose–response experiment 2 h |
| Krejci et al., 2007 [47] | 180 min |
| Lima et al., 2012 [62] | 1 h |
| Luckner et al., 2006 [27] | Not reported |
| Marcus et al., 2026 [55] | 90 min |
| Morelli et al., 2011 [28] | 6 h |
| Morelli et al., 2011 [32] | Single TP bolus |
| Nascente et al., 2017 [30] | 60 min |
| Nakajima et al., 2006 [57] | 60 min |
| Nygren et al., 2009 [31] | 30 min per dose |
| Qiu et al., 2014 [58] | Continuous TP infusion. Duration not reported; to restore baseline MAP |
| Ristagno et al., 2007 [64] | Single AVP bolus |
| Saeki et al, 1991 [33] | Rats: 20 min  Human: 10 min |
| Schulz et al., 2021 [54] | 30 min per dose, at least 90 min |
| Truse et al., 2019 [44] | Continuous AVP infusion. Dose increased every 30 min with measurements 30 min after increasing. |
| Van Loon et al., 2020 [26] | 5 h |
| Wafa et al., 2015 [50] | Single DDAVP bolus |
| Westphal et al., 2004 [48] | AVP infusion to achieve increases in MAP: 15 ± 5 min to increase MAP by 20 mmHg and 30 ± 5 min to increase MAP by 40 mmHg. |
| Wiles et al., 1986 [29] | 90 min |
