# Supplementary material for: Effect of vasopressin and its analogues on microcirculatory perfusion: a systematic review
Source: Intensive Care Med Exp. 2026 Oct 10;14:132. doi: 10.1186/s40635-026-00983-5 (PMC13653856; doi:10.1186/s40635-026-00983-5)
Supplement: Supplementary file 6 [file 40635_2026_983_MOESM6_ESM.docx]

**Additional file 7: risk of bias assessment**

| **Table S7.1** Risk of bias assessment of randomized in vivo animal studies using the SYRCLE risk of bias tool. | | | | | | | | | | | |
| --- | --- | --- | --- | --- | --- | --- | --- | --- | --- | --- | --- |
| **Study** | **Sequence generation (selection bias)** | **Baseline characteristics (selection bias)** | **Allocation concealment (selection bias)** | **Random housing (performance bias)** | **Blinding (performance bias)** | **Random outcome assessment (detection bias)** | **Blinding (detection bias)** | **Incomplete outcome data (attrition bias)** | **Selective outcome reporting (reporting bias)** | **Other source of bias (other)** | **Overall risk of bias** |
| Albert *et al.*, 2004 [59] | Low | Low | Unclear | Unclear | Unclear | Low | Unclear | High | Low | Low | 6/20 |
| Asfar *et al.*, 2003 [53] | Low | Low | Unclear | Unclear | Low | Unclear | Low | Low | Low | Low | 3/20 |
| Bomberg *et al.*, 2013 [49] | Low | Low | Unclear | Unclear | Unclear | Low | Unclear | Unclear | Low | Low | 5/20 |
| Bomberg *et al.*, 2014 [45] | Low | Low | Unclear | Unclear | Unclear | Low | Unclear | Low | Low | Low | 4/20 |
| Cheung et al., 2007 [60] | Low | Low | Unclear | Unclear | Unclear | Low | Unclear | Low | Low | High | 6/20 |
| Friesenecker *et al.*, 2004 [37] | Low | Low | Unclear | Low | Unclear | Low | Unclear | Low | Low | Low | 3/20 |
| Friesenecker *et al.*, 2006 [43] | Low | Low | Unclear | Unclear | Unclear | Unclear | Unclear | Low | Low | Low | 5/20 |
| Gonzalez *et al.*, 2016 [61] | Low | Low | Unclear | Unclear | Unclear | Low | Low | Low | Low | High | 5/20 |
| Hiltebrand *et al.*, 2007 [52] | Low | Low | Unclear | Unclear | Unclear | Low | Unclear | Unclear | Low | Low | 5/20 |
| Jufar *et al.*, 2026 [51] | Low | Low | Unclear | Unclear | High | Low | High | High | Low | High | 10/20 |
| Kildal *et al.,* 2015 [36] | Unclear | Low | Unclear | Unclear | Unclear | Low | Unclear | Unclear | Low | High | 8/20 |
| Klein *et al.*, 2021 [63] | Low | Low | Unclear | Unclear | High | Low | High | Low | Low | High | 8/20 |
| Knotzer *et al.*, 2005 [35] | Low | Low | Unclear | Unclear | Unclear | Low | Unclear | Unclear | Low | Unclear | 6/20 |
| Krejci *et al.*, 2007 [47] | Low | Low | Low | Unclear | Unclear | Unclear | Unclear | Unclear | Low | Low | 5/20 |
| Lima *et al.*, 2012 [62] | Low | Low | Unclear | Unclear | Unclear | Unclear | High | Low | Low | Low | 6/20 |
| Marcus *et al.,* 2026 [55] | Low | Low | Unclear | Unclear | Low | Low | Unclear | Unclear | Low | Low | 4/20 |
| Nakajima *et al.*, 2006 [57] | Low | Low | Unclear | Unclear | Unclear | Unclear | Unclear | Unclear | Unclear | Unclear | 8/20 |
| Qiu *et al.*, 2014 [58] | Low | Low | Unclear | Unclear | Unclear | Low | Low | Low | Low | Unclear | 4/20 |
| Ristagno *et al.*, 2007 [64] | Low | Low | Unclear | Unclear | Low | Low | Low | Low | Low | High | 4/20 |
| Schulz *et al.*, 2021 [54] | Low | Low | Unclear | Unclear | Low | Low | Low | Low | Low | Low | 2/20 |
| Truse *et al.*, 2019 [44] | Low | Unclear | Low | Low | Low | Low | Low | Low | Unclear | Low | 2/20 |
| Wafa *et al.*, 2015 [50] | Low | Unclear | Unclear | Unclear | Low | Low | Low | Low | Low | Low | 3/20 |
| Westphal *et al.*, 2004 [48] | Low | Unclear | Unclear | Low | Low | Low | Low | High | Low | Low | 4/20 |

| **Table S7.2** Risk of bias assessment of non-randomized in vivo animal studies using the SYRCLE risk of bias tool | | | | | | | | | | | |
| --- | --- | --- | --- | --- | --- | --- | --- | --- | --- | --- | --- |
| **Study** | **Sequence generation (selection bias)** | **Baseline characteristics (selection bias)** | **Allocation concealment (selection bias)** | **Random housing (performance bias)** | **Blinding (performance bias)** | **Random outcome assessment (detection bias)** | **Blinding (detection bias)** | **Incomplete outcome data (attrition bias)** | **Selective outcome reporting (reporting bias)** | **Other sources of bias (other)** | **Overall risk of bias** |
| Correia *et al.*, 2001 [38] | High | Low | Unclear | Unclear | Low | Low | Low | Low | Low | Unclear | 5/20 |
| Cowley *et al.*, 1998 [39] | High | Low | Unclear | Low | Unclear | Unclear | Low | Low | Low | Low | 5/20 |
| Evans *et al.*, 2000 [40] | High | Low | Unclear | Unclear | Unclear | High | Unclear | High | Low | Low | 10/20 |
| Faivre *et al.*, 2005 [46] | High | Low | Unclear | Unclear | Unclear | Unclear | Low | Unclear | Low | Low | 7/20 |
| Franchini *et al.*, 1996 [41] | High | High | Unclear | Unclear | Unclear | Unclear | Unclear | Low | Low | High | 11/20 |
| Franchini *et al.*, 1997 [42] | High | Low | High | Unclear | Unclear | Unclear | Unclear | Unclear | Low | Unclear | 10/20 |
| Holliger *et al.*, 1983 [34] | High | High | Unclear | Unclear | Unclear | Unclear | Unclear | Unclear | Low | Unclear | 11/20 |
| Kopel *et al.*, 2008 [56] | High | Low | Unclear | Unclear | Unclear | Unclear | Unclear | Low | Low | Unclear | 8/20 |
| Saeki *et al.*, 1991 [33] | High | Unclear | High | Unclear | Unclear | Unclear | High | Unclear | Low | High | 13/20 |

| **Table S7.3** Risk of bias assessment of randomized human studies using RoB 2 tool | | | |
| --- | --- | --- | --- |
| **Criteria** | **van Loon *et al.* 2020 [26]** | **Luckner *et al.* 2006 [27]** | **Morelli *et al.* 2011 [28]** |
| Domain 1: Risk of bias arising from the randomization process | Some concerns | Low risk | Some concerns |
| Domain 2: Risk of bias due to deviations from the intended interventions | Low risk | Some concerns | Some concerns |
| Domain 3: Risk of bias due to missing outcome data | Low risk | Some concerns | Low risk |
| Domain 4: Risk of bias in measurement of the outcome | Low risk | NA | Low risk |
| Domain 5: Risk of bias in selection of reported result | Some concerns | Some concerns | Low risk |

NA: not assessed; this study did not report MFI: microvascular flow index, outcome-measurement domain was evaluated for MFI only.

| **Table S7.4** Risk of bias assessment of included prospective human studies using the ROBINS-I tool | | | | | | | |
| --- | --- | --- | --- | --- | --- | --- | --- |
| **Study** | **Bias due to confounding** | **Bias in selection of participants** | **Bias in measurement of exposure** | **Bias due to missing data** | **Bias in measurement of outcomes** | **Bias due to selective reporting** | **Overall risk of bias** |
| Morelli *et al*. 2011 [32] | Serious | Moderate | Moderate | Low | Low | Low | Serious |
| Nascente *et al.* 2017 [30] | Serious | Moderate | Low | Low | NA | Moderate | Serious^1^ |
| Nygren et al. 2009 [31] | Serious | Moderate | Low | Low | NA | Low | Serious^1^ |
| Wiles et al. 1986 [29] | Moderate | Low | Low | Low | NA | Moderate | Moderate^1^ |

NA: not assessed; this study did not report MFI: microvascular flow index, outcome-measurement domain was evaluated for MFI only.

^1^ The overall judgement is based on the remaining six domains; the outcome-measurement domain was not assessed (MFI not reported).

| **Table S7.5** Risk of bias assessment of included observational human studies using the NOS tool | | | | | |
| --- | --- | --- | --- | --- | --- |
| **Study** | **Design** | **Selection (0–4★)** | **Comparability (0–2★)** | **Outcome (0–3★)** | **Total NOS-score (0–9★)** |
| Saeki et al. 1991 [33] | Observational cohort | 3★ | 0★ | 1★ | **4★** |

**Table S7.6 Direction of microcirculatory perfusion measurements stratified by risk of bias**

| **Analysis** | **Lower risk of bias** |  |  | **Higher risk of bias** |  |  |
| --- | --- | --- | --- | --- | --- | --- |
|  | **Measurements** | **n ↓/↔/↑** | **% ↓/↔/↑** | **Measurements** | **n ↓/↔/↑** | **% ↓/↔/↑** |
| A. Animal study-units (SYRCLE ≤ 5 vs > 5) |  |  |  |  |  |  |
| All measurements | 80 | 28/37/15 | 35/46/19 | 46 | 19/19/8 | 41/41/17 |
| By haemodynamic condition |  |  |  |  |  |  |
| Physiological | 29 | 12/15/2 | 41/52/7 | 17 | 14/3/0 | 82/18/0 |
| Vasodilatory / septic shock | 48 | 16/21/11 | 33/44/23 | 20 | 4/12/4 | 20/60/20 |
| Haemorrhagic shock | 1 | 0/1/0 | 0/100/0 | 5 | 1/0/4 | 20/0/80 |
| Cardiac dysfunction / arrest | 2 | 0/0/2 | 0/0/100 | 4 | 0/4/0 | 0/100/0 |
| By vascular bed |  |  |  |  |  |  |
| Gastrointestinal | 51 | 15/23/13 | 29/45/25 | 12 | 4/5/3 | 33/42/25 |
| Renal cortex | 5 | 2/3/0 | 40/60/0 | 6 | 1/4/1 | 17/67/17 |
| Renal medulla | 3 | 2/1/0 | 67/33/0 | 10 | 8/2/0 | 80/20/0 |
| Sublingual | 1 | 0/1/0 | 0/100/0 | 9 | 5/4/0 | 56/44/0 |
| Cutaneous | 18 | 9/9/0 | 50/50/0 | 4 | 0/0/4 | 0/0/100 |
| Cerebral | 2 | 0/0/2 | 0/0/100 | 4 | 0/4/0 | 0/100/0 |
| Conjunctival | 0 | – | – | 1 | 1/0/0 | 100/0/0 |
| B. Human study-units (mapped categories, see footnote) |  |  |  |  |  |  |
| All measurements | 14 | 1/13/0 | 7/93/0 | 12 | 2/4/6 | 17/33/50 |
| By haemodynamic condition |  |  |  |  |  |  |
| Physiological | 1 | 1/0/0 | 100/0/0 | 0 | – | – |
| Vasodilatory / septic shock | 13 | 0/13/0 | 0/100/0 | 10 | 2/4/4 | 20/40/40 |
| Cirrhosis-related portal hypertension | 0 | – | – | 2 | 0/0/2 | 0/0/100 |
| By vascular bed |  |  |  |  |  |  |
| Gastrointestinal | 0 | – | – | 3 | 1/0/2 | 33/0/67 |
| Sublingual | 12 | 0/12/0 | 0/100/0 | 9 | 1/4/4 | 11/44/44 |
| Cutaneous | 2 | 1/1/0 | 50/50/0 | 0 | – | – |

Sensitivity analysis of the direction of individual microcirculatory perfusion measurements, stratified by risk of bias. Percentages are of measurements within each stratum and may not sum to 100 owing to rounding. Panel A: animal study-units, all assessed with the SYRCLE tool. As SYRCLE does not define a threshold for overall risk of bias, study-units were dichotomised at the median score of the included animal publications (5 of a possible 20; lower scores indicate lower risk of bias). Panel B: human study-units, assessed with three different instruments. To permit stratification, judgements were mapped onto a common two-level scale: RoB 2 "low risk" or "some concerns" and ROBINS-I "low" or "moderate" were assigned to the lower stratum, and RoB 2 "high risk", ROBINS-I "serious" or "critical", and a Newcastle-Ottawa score below 5 of 9 stars to the higher stratum. This mapping reflects the correspondence between RoB 2 and ROBINS-I categories described by the tool developers, but is a judgement made for this review; the small number of human study-units means the human analysis is exploratory.

**Table S7.7 Overall direction of effect of each study-unit by risk-of-bias stratum**

| **Direction of effect** | **Animal study-units** |  |  | **Human study-units** |  |  |
| --- | --- | --- | --- | --- | --- | --- |
|  | **Total** | **Lower risk** | **Higher risk** | **Total** | **Lower risk** | **Higher risk** |
| Decreased | 10 | 4 | 6 | 2 | 1 | 1 |
| Unchanged | 6 | 3 | 3 | 4 | 3 | 1 |
| Increased | 2 | 1 | 1 | 2 | 0 | 2 |
| Mixed | 17 | 11 | 6 | 0 | 0 | 0 |
| All study-units | 35 | 19 | 16 | 8 | 4 | 4 |

Cross-tabulation of the overall direction of effect of each study-unit against risk-of-bias stratum. A study-unit was classified as decreased, unchanged or increased when all reported perfusion parameters changed in the same direction, and as mixed otherwise; the classification rule is given in the Methods. Strata are defined in the footnote to Table S7.6.

**Table S7.8 Risk of bias and direction of effect for each study-unit**

| **Study [ref]** | **Design** | **Tool** | **Judgement or score** | **Stratum** | **Haemodynamic condition** | **Vascular bed** | **Measurements (n)** | **Direction of effect** |
| --- | --- | --- | --- | --- | --- | --- | --- | --- |
| Schulz 2021 [54] | Randomised, blinded with repeated-measures dose escalation | SYRCLE | 2/20 | Lower | Vasodilatory / septic shock | Gastrointestinal | 10 | Mixed |
| Truse 2019 [44] | Randomised, blinded crossover | SYRCLE | 2/20 | Lower | Physiological | Gastrointestinal | 5 | Mixed |
| Truse 2019 [44] | Randomised, blinded crossover | SYRCLE | 2/20 | Lower | Physiological | Cutaneous | 11 | Mixed |
| Asfar 2003 [53] | RCT | SYRCLE | 3/20 | Lower | Vasodilatory / septic shock | Gastrointestinal | 2 | Mixed |
| Friesenecker 2004 [37] | RCT | SYRCLE | 3/20 | Lower | Physiological | Cutaneous | 3 | Decreased |
| Wafa 2015 [50] | RCT | SYRCLE | 3/20 | Lower | Vasodilatory / septic shock | Gastrointestinal | 2 | Unchanged |
| Bomberg 2014 [45] | RCT | SYRCLE | 4/20 | Lower | Vasodilatory / septic shock | Gastrointestinal | 1 | Decreased |
| Marcus 2026 [55] | RCT | SYRCLE | 4/20 | Lower | Vasodilatory / septic shock | Gastrointestinal | 8 | Mixed |
| Qiu 2014 [58] | RCT | SYRCLE | 4/20 | Lower | Vasodilatory / septic shock | Gastrointestinal | 8 | Mixed |
| Ristagno 2007 [64] | RCT | SYRCLE | 4/20 | Lower | Cardiac dysfunction / arrest | Cerebral | 2 | Increased |
| Westphal 2004 [48] | RCT | SYRCLE | 4/20 | Lower | Vasodilatory / septic shock | Gastrointestinal | 1 | Decreased |
| Bomberg 2013 [49] | RCT | SYRCLE | 5/20 | Lower | Vasodilatory / septic shock | Gastrointestinal | 2 | Unchanged |
| Correia 2001 [38] | Non-randomised in vivo animal study | SYRCLE | 5/20 | Lower | Physiological | Renal | 2 | Mixed |
| Cowley 1998 [39] | Non-randomised in vivo animal study | SYRCLE | 5/20 | Lower | Physiological | Renal | 4 | Mixed |
| Friesenecker 2006 [43] | RCT | SYRCLE | 5/20 | Lower | Physiological | Cutaneous | 4 | Mixed |
| González 2016 [61] | RCT | SYRCLE | 5/20 | Lower | Haemorrhagic shock | Sublingual | 1 | Unchanged |
| Hiltebrand 2007 [52] | RCT | SYRCLE | 5/20 | Lower | Vasodilatory / septic shock | Gastrointestinal | 8 | Mixed |
| Krejci 2007 [47] | RCT | SYRCLE | 5/20 | Lower | Vasodilatory / septic shock | Gastrointestinal | 4 | Mixed |
| Krejci 2007 [47] | RCT | SYRCLE | 5/20 | Lower | Vasodilatory / septic shock | Renal | 2 | Decreased |
| Albert 2004 [59] | RCT | SYRCLE | 6/20 | Higher | Vasodilatory / septic shock | Renal | 4 | Mixed |
| Cheung 2007 [60] | RCT | SYRCLE | 6/20 | Higher | Haemorrhagic shock | Conjunctival | 1 | Decreased |
| Knotzer 2005 [35] | RCT | SYRCLE | 6/20 | Higher | Physiological | Gastrointestinal | 1 | Decreased |
| Lima 2012 [62] | RCT | SYRCLE | 6/20 | Higher | Haemorrhagic shock | Cutaneous | 4 | Increased |
| Faivre 2005 [46] | Non-randomised in vivo animal study | SYRCLE | 7/20 | Higher | Vasodilatory / septic shock | Renal | 2 | Decreased |
| Kildal 2015 [36] | RCT | SYRCLE | 8/20 | Higher | Physiological | Sublingual | 5 | Decreased |
| Klein 2021 [63] | RCT | SYRCLE | 8/20 | Higher | Cardiac dysfunction / arrest | Sublingual | 4 | Unchanged |
| Kopel 2008 [56] | Non-randomised in vivo animal study | SYRCLE | 8/20 | Higher | Vasodilatory / septic shock | Gastrointestinal | 3 | Mixed |
| Nakajima 2006 [57] | RCT | SYRCLE | 8/20 | Higher | Vasodilatory / septic shock | Gastrointestinal | 5 | Mixed |
| Evans 2000 [40] | Non-randomised repeated-measures in vivo animal study | SYRCLE | 10/20 | Higher | Physiological | Renal | 2 | Mixed |
| Franchini 1997 [42] | Non-randomised in vivo animal study | SYRCLE | 10/20 | Higher | Physiological | Renal | 2 | Mixed |
| Jufar 2026 [51] | RCT | SYRCLE | 10/20 | Higher | Vasodilatory / septic shock | Cerebral | 4 | Unchanged |
| Jufar 2026 [51] | RCT | SYRCLE | 10/20 | Higher | Vasodilatory / septic shock | Renal | 2 | Unchanged |
| Franchini 1996 [41] | Non-randomised in vivo animal study | SYRCLE | 11/20 | Higher | Physiological | Renal | 4 | Mixed |
| Holliger 1983 [34] | Non-randomised in vivo animal study | SYRCLE | 11/20 | Higher | Physiological | Gastrointestinal | 2 | Decreased |
| Saeki 1991 (animals) [33] | Observational study | SYRCLE | 13/20 | Higher | Physiological | Gastrointestinal | 1 | Decreased |
| Luckner 2006 [27] | RCT | RoB 2 | Some concerns | Lower | Vasodilatory / septic shock | Cutaneous | 1 | Unchanged |
| Morelli 2011 (Crit Care) [28] | RCT | RoB 2 | Some concerns | Lower | Vasodilatory / septic shock | Sublingual | 10 | Unchanged |
| Wiles 1986 [29] | Prospective study | ROBINS-I | Moderate | Lower | Physiological | Cutaneous | 1 | Decreased |
| van Loon 2020 [26] | RCT | RoB 2 | Some concerns | Lower | Vasodilatory / septic shock | Sublingual | 2 | Unchanged |
| Morelli 2011 (Intensive Care Med) [32] | Prospective study | ROBINS-I | Serious | Higher | Vasodilatory / septic shock | Sublingual | 5 | Increased |
| Nascente 2017 [30] | Prospective study | ROBINS-I | Serious | Higher | Vasodilatory / septic shock | Sublingual | 4 | Unchanged |
| Nygren 2009 [31] | Prospective study | ROBINS-I | Serious | Higher | Vasodilatory / septic shock | Gastrointestinal | 1 | Decreased |
| Saeki 1991 (humans) [33] | Observational study | NOS | 4 of 9 stars | Higher | Cirrhosis-related portal hypertension | Gastrointestinal | 2 | Increased |

Risk of bias, haemodynamic condition, vascular bed and direction of effect for each of the 43 study-units, ordered by species and risk-of-bias stratum. Strata are defined in the footnote to Table S7.6.
