# Supplementary material for: Effect of vasopressin and its analogues on microcirculatory perfusion: a systematic review
Source: Intensive Care Med Exp. 2026 Oct 10;14:132. doi: 10.1186/s40635-026-00983-5 (PMC13653856; doi:10.1186/s40635-026-00983-5)
Supplement: Supplementary file 7 [file 40635_2026_983_MOESM7_ESM.docx]

**Additional file 8: perfusion details**

| Table S8.1: Effect of AVP on microcirculatory perfusion in normal physiological conditions in animal and humans | |
| --- | --- |
| Study | Effect (intervention vs control/before vs after) |
| Holliger et al., 1983 [34] | - AVP **vs** saline   ↓ RBC and ↓ flow index (dose-dependent; significant at ≥10 mU/kg; *P* < 0.05) |
| Knotzer et al., 2005 [35] | - AVP **vs** saline   ↓ jejunal microvascular blood flow (133 → 82 PU) |
| Saeki et al., 1991 animals [33] | - AVP **vs** baseline   ↓ GMBF (37%) |
| Truse et al., 2019 [44] | - 0.001 ng/kg/min AVP **vs** baseline   ↑ Gastric μflow (149 ± 79 → 237 ± 78 aU *P* < 0.05)  ↔ Gastric μvelo   - 0.01 ng/kg/min AVP **vs** baseline   ↑ Gastric μflow (149 ± 79 → 221 ± 87 aU *P* < 0.05)  ↔ Gastric μvelo   - 1 ng/kg/min AVP **vs** baseline   ↓ Gastric μflow *P* < 0.05 |
| Correia et al., 2001 [38] | - V1a agonist **vs** baseline   ↔ Cortical blood flow  ↓ Medullary blood flow 30 ± 8% (*P* = 0.02) |
| Cowley et al., 1998 [39] | - AVP **vs** control   ↔ Cortical blood flow  ↔ Medullary blood flow ~↓13% (NS)   - V1a agonist **vs** control   ↔ Cortical blood flow  ↓ Medullary blood flow 35–40% (*P* < 0.05) |
| Evans et al., 2000 [40] | - V1a agonist **vs** control   ↔ Cortical blood flow  ↓ Medullary blood flow 41 ± 7 % (*P* ≤ 0.001) |
| Franchini et al., 1996 [41] | - AVP **vs** saline   ↔ Cortical blood flow  ↓ Inner medullary blood flow ~10% 0.065 ng/kg/min (*P* < 0.05)  ↓ Inner medullary blood flow ~21% 0.25 ng/kg/min (*P* < 0.05)  ↓ Inner medullary blood flow ~26% 0.50 ng/kg/min (*P* < 0.05) |
| Franchini et al., 1997 [42] | - AVP **vs** baseline   ↔ Cortical blood flow  ↓ Inner medullary blood flow ~24% (*P* < 0.05) |
| Kildal et al., 2015 [36] | - AVP **vs** baseline   0.003 IU/kg/min AVP:  ↓ MFI 2.77 ± 0.04 → 2.03 ± 0.38 (P < 0.05)  0.006 IU/kg/min AVP:  ↓ MFI (P < 0.05)  0.012 IU/kg/min AVP:  ↓ MFI (P < 0.05)   - AVP in acute heart failure **vs** AVP in pre-ischemic state   0.001 IU/kg/min AVP:  ↓ MFI 1.62 ± 0.60 vs 2.86 ± 0.09 (P < 0.05)  0.003 IU/kg/min AVP:  ↓ MFI 1.16 ± 0.38 vs 2.03 ± 0.38 (P < 0.05) |
| Friesenecker et al., 2004 [37] | - AVP **vs** baseline   ↓ Microvascular blood flow  ↓ FCD (0.62 ± 0.70 of baseline; *P* < 0.05)  ↓ RBC velocity |
| Friesenecker et al., 2006 [43] | - AVP **vs** NE   ↓ A0 arteriolar blood flow (-80% vs -62% *P* = 0.004)  ↔ A0 RBC velocity  ↔ A1-A4 arteriolar blood flow  ↔ A1-A4 RBC velocity |
| Truse et al., 2019 [44] | - 0.001 ng/kg/min AVP **vs** saline   ↔ Oral μflow  ↔ Oral μvelo   - 0.01 ng/kg/min AVP **vs** saline   ↔ Oral μflow  ↔ Oral μvelo   - 0.1 ng/kg/min AVP **vs** saline   ↔ Oral μflow   - 1 ng/kg/min AVP **vs** saline   ↓ Oral μflow (122 ± 49 → 53 ± 25 aU *P* < 0.05)  ↔ Oral μvelo (22 ± 6 → 16 ± 2 aU)  ↓ TVD (21 ± 1.3 → 18 ± 2.7 mm/mm² *P* < 0.05)  ↓ PVD (*P* < 0.05)  ↓ PPV (61 ± 11% → 32 ± 14.6% *P* < 0.05)  ↓ MFI (*P* < 0.05) |
| Wiles et al., 1986 [29] | - AVP **vs** baseline   ↓ Blood flow (2.6 → 2.3 V of baseline *P* < 0.001) |

| Table S8.2: Effect of AVP in ****microcirculation in vasodilatory / septic shock**** | |
| --- | --- |
| Study | Effect (intervention vs control/before vs after) |
| Asfar et al., 2003 [53] | - Terlipressin + LPS **vs** LPS   ↓IPI (*P* <0.05)   - Terlipressin + LPS + fluid challenge **vs** LPS + fluid challenge   ↑ IPI (*P* <0.05) |
| Bomberg et al., 2013 [49] | - CPB + AVP **vs** baseline/sham   ↔ AU *(P* > 0.05)  ↔ FCD 6-13% *P* = 0.1 |
| Bomberg et al., 2014 [45] | - AVP +CPB **vs** CPB ≠ AVP   ↓ AU (~ 26.5 vs 75.5 AU; P < 0.001) |
| Hiltebrand et al., 2007 [52] | - AVP **vs** crystalloids in sepsis   ↓ gastric mucosal MBF (~23; *P* < 0.05)  ↓ gastric muscularis MBF (~48; *P* < 0.05  ↓ jejunal mucosal MBF (~27 *P* < 0.05)  ↔ jejunal muscularis MBF *(P* > 0.05)  ↔ colonic mucosal MBF *(P* > 0.05)  ↓ colonic muscularis MBF (~22; *P* < 0.05)   - AVP **vs** crystalloids without sepsis   ↓ microcirculatory blood flow in all gastrointestinal regions (*P* ≤ 0.05) |
| Kopel et al., 2008 [56] | - AVP + endotoxic shock **vs** AVP + saline bolus without endotoxins   Liver: ↔  Gut 1-100 ng: ↑  Gut >100 ng: ↔ |
| Krejci et al., 2007 [47] | - AVP **vs** baseline in sepsis   Pancreas:↓ MBF 36 ± 14% (*P* < 0.01)  Liver: ↔ MBF   - AVP **vs** baseline without sepsis   Pancreas: ↓ MBF ~74–76% (*P* < 0.05)  Liver: ↔ MBF |
| Marcus et al., 2026 [55] | - AVP + milrinon **vs** baseline in sepsis   Liver: ↔ µFlow  Colon: ↑ µFlow (*P* < 0.05)   - AVP + levosimendan **vs** baseline in sepsis   Liver: ↔ µFlow  Colon: ↑ µFlow (*P* < 0.05)   - AVP + milrinon **vs** baseline without sepsis   Liver: ↔ µFlow  Colon: ↔ µFlow   - AVP + levosimendan **vs** baseline without sepsis   Liver: ↔ µFlow  Colon: ↔ µFlow |
| Nakajima et al., 2006 [57] | - AVP **vs** saline   RBC flux:↔  Velocity: ↔  PVD: ↔   - AVP + L-arginine **vs** AVP alone   RBC flux & velocity: ↑ vs AVP alone (*P* < 0.005–0.001)  PVD: ↑ greater preservation vs AVP alone (*P* < 0.001) |
| Qiu et al., 2014 [58] | - TP vs 0.5 mL saline   ↑ MFI, restored to baseline (*P* < 0.05)  ↑ PPV, restored to baseline (*P* < 0.05)  ↓ HI, restored to baseline (*P* < 0.05)  ↓ PVD p > 0.05   - TP vs NE   ↑ MFI TP vs NE (*P* < 0.05)  ↓ HI TP vs NE (*P* < 0.05)  ↔ PVD (*P* > 0.05)  ↔ PPV (*P* > 0.05) |
| Schulz et al., 2021 [54] | - Sub-therapeutic AVP **vs** baseline in mild sepsis   ↑ TVD (+4.8 ± 5.2 mm/mm²; *P* < 0.05)  ↔ PVD   - Therapeutic AVP **vs** baseline in mild sepsis   ↔ TVD  ↔ PVD   - Sub-therapeutic AVP **vs** baseline & **vs** vehicle in moderate sepsis   ↑ TVD (+5.2 ± 3.0 mm/mm² **vs** baseline), ↑ **vs** vehicle (*P* < 0.05)  ↑ PVD *P* < 0.05 **vs** vehicle   - Therapeutic AVP **vs** vehicle in moderate sepsis   ↔ TVD  ↔ PVD   - Sub-therapeutic AVP **vs** therapeutic AVP in moderate sepsis   ↑ TVD (*P* <0.05)  ↑ PVD (*P* <0.05) |
| Wafa et al., 2015 [50] | - DDAVP **vs** saline   FCD: ↔   - DDAVP + LPS **vs** LPS   FCD: ↔ |
| Westphal et al., 2004 [48] | - AVP **vs** saline:   ↓ RBC velocity (−45% 🡪 −47%, *P* < 0.05) |
| Albert et al., 2004 [59] | - AVP bolus **vs** baseline in sepsis   ↑ Cortical blood flow (*P* < 0.05)  ↔ Medullary blood flow   - AVP bolus **vs** baseline without sepsis   ↓ Cortical blood flow (*P* < 0.001)  ↓ Medullary blood flow (*P* < 0.05) |
| Faivre et al., 2005 [46] | - AVP vs saline   ↓ Medullary PU (*P* < 0.05)   - AVP + levosimendan vs saline   ↓ Medullary PU (P < 0.05) |
| Jufar et al., 2026 [51] | - AVP **vs** pre-infusion control (CPB baseline)   ↔ Cortical perfusion  ↔ Medullary perfusion   - AVP vs baseline during CPB   ↔ Cerebral PU   - AVP **vs** NE   ↔ Cerebral PU   - AVP **vs** metaraminol   ↔ Cerebral PU   - AVP **vs** phenlypehrine   ↔ Cerebral PU |
| Krejci et al., 2007 [47] | - AVP **vs** Ringer Lactate in sepsis   ↓ 16 ± 20% (*P* < 0.01)   - AVP **vs** Ringer Lactate without sepsis   ↓ (106 ± 15 → 86 ± 16%; *P* < 0.01) |
| Nygren et al., 2009 [31] | - AVP + NE **vs** NE   ↓ PU (AVP + NE: 210 PU, NE: 264 ; *P* < 0.01) |
| Morelli et al., 2011 [28] | - TP + NE **vs** NE   ↔ MFI  ↔ PVD  ↔ PPV  ↔ TVD  ↔ HI   - AVP + NE **vs** NE   ↔ MFI  ↔ PVD  ↔ PPV  ↔ TVD  ↔ HI |
| Morelli et al., 2011 [32] | - TP + NE **vs** baseline   ↑ MFI (P < 0.05)  ↑ PVD (P < 0.05)  ↑ PPV (P < 0.05)  ↑ TVD (P < 0.05)  ↓ HI (P < 0.05) |
| Nascente et al., 2017 [30] | - AVP + NE **vs** NE   ↔ MFI  ↔ TVD  ↔ PVD  ↔ PPV |
| Van Loon et al., 2020 [26] | - AVP **vs** saline   ↔ TVD  ↔ MFI |
| Luckner et al., 2006 [27] | - AVP + NE **vs** NE   ↔ pre-ischemic AUC |

| Table S8.3: Effect of ****AVP on microcirculation in hemorrhagic shock / resuscitation models**** | |
| --- | --- |
| Study | Effect (intervention vs control/before vs after) |
| Cheung et al., 2007 [60] | - AVP (post-resuscitation phase) **vs** pre-resuscitation phase   ↓ RBC velocity (~70%) |
| Gonzalez et al., 2016 [61] | - Post-resuscitation **vs** pre-resuscitation phase   ↑ PVD (p < 0.05)  ↑ MFI (p < 0.001)  ↓ HI (p < 0.05)  - Resuscitation treatment (saline **vs** albumin + hypertonic saline **vs** albumin + hypertonic saline + TP  ↔ |
| Lima et al., 2012 [62] | - AVP **vs** saline resuscitation   ↑ FCD (P < 0.005)  ↑ RBC velocity (P < 0.005)   - AVP **vs** NE (post-resuscitation)   ↑ FCD (*P* < 0.005)  ↑ RBC velocity (*P* < 0.005) |

| Table S8.4: ****Effect of AVP on microcirculation in cardiac dysfunction and arrest**** | |
| --- | --- |
| Study | Effect (intervention vs control/before vs after) |
| Klein et al., 2021 [63] | - AVP **vs** NE   ↔ TVD  ↔ PVD  ↔ PPV  ↔ MFI |
| Ristagno et al., 2007 [64] | - AVP **vs** epinephrine   ↑ MBF index (*P* < 0.05–0.01)  ↑ Number of perfused microvessels *P* < 0.01 |

| Table S8.5: Effect of AVP on microcirculation in cirrhosis-related portal hypertension | |
| --- | --- |
| Study | Effect (intervention vs control/before vs after) |
| Saeki et al. 1991 human [33] | - AVP **vs** baseline   Antrum: ↑ GMBF 14/22 patients  Gastric body: ↑ GMBF 19/24 patients |
