# Supplementary material for: Effect of vasopressin and its analogues on microcirculatory perfusion: a systematic review
Source: Intensive Care Med Exp. 2026 Oct 10;14:132. doi: 10.1186/s40635-026-00983-5 (PMC13653856; doi:10.1186/s40635-026-00983-5)
Supplement: Supplementary file 8 [file 40635_2026_983_MOESM8_ESM.docx]

Does vasopressin help or harm the microcirculation? Our systematic review of 39 studies and 152 measurements finds there is no single answer. The effect depends on the haemodynamic condition, the vascular bed, volume status and the comparator used. Perfusion tended to fall under physiological conditions, but in vasodilatory or septic shock the picture was heterogeneous and sometimes favourable. The renal medulla showed the highest proportion of reductions, though almost all of this evidence is preclinical, and no human study has yet assessed renal microcirculation. #microcirculation #vasopressin #septicshock
