# Supplementary material for: Effect of vasopressin and its analogues on microcirculatory perfusion: a systematic review
Source: Intensive Care Med Exp. 2026 Oct 10;14:132. doi: 10.1186/s40635-026-00983-5 (PMC13653856; doi:10.1186/s40635-026-00983-5)
Supplement: Supplementary file 9 [file 40635_2026_983_MOESM9_ESM.docx]

**Additional file 4: definitions, abbreviations, and units of microcirculatory perfusion parameters**

|  | | | |
| --- | --- | --- | --- |
| **Abbreviation** | **Parameter** | **Assessment method** | **Unit** |
| **TVD** | Total vessel density | Videomicroscopy (IDF/SDF/OPS) | mm/mm² |
| **PVD** | Perfused vessel density | Videomicroscopy (IDF/SDF/OPS) | mm/mm² |
| **PPV** | Proportion of perfused vessels | Videomicroscopy (IDF/SDF/OPS) | % |
| **FCD** | Functional capillary density | Videomicroscopy (IDF/SDF/OPS) | mm/mm² |
| **RBC velocity** | Red blood cell velocity | Videomicroscopy (IDF/SDF/OPS) | µm/s |
| **RBC flux** | Red blood cell flux | Videomicroscopy (IDF/SDF/OPS) | cells/s |
| **MFI** | Microvascular flow index | Videomicroscopy (IDF/SDF/OPS) | Score 0–3 |
| **HI** | Heterogeneity index | Videomicroscopy (IDF/SDF/OPS) | Dimensionless |
| **(B)PU** | (Blood) perfusion units | Laser Doppler | PU |
| **V** | Voltage units | Laser Doppler | V |
|  | Skin blood flow | Laser Doppler | V |
| **IPI** | Ileal perfusion index | Laser Doppler | Index |

(B)PU, (blood) perfusion units; FCD, functional capillary density; HI, heterogeneity index; IDF, incident dark field; IPI, ileal perfusion index; MFI, microvascular flow index; OPS, orthogonal polarisation spectral; PPV, proportion of perfused vessels; PVD, perfused vessel density; RBC, red blood cell; SDF, sidestream dark field; TVD, total vessel density; V, voltage.
